# Supplementary material for: An in vitro study in separating tensile loads during maxillo-mandibular fixation using wire and/or elastics
Source: PLoS One. 2024 Mar 15;19(3):e0300481. doi: 10.1371/journal.pone.0300481 (PMC10942067; doi:10.1371/journal.pone.0300481)
Supplement: S1 Fig — Data collected from pilot study. (PDF) [file pone.0300481.s001.pdf]

INTERLOCKING+OVERLAPPING ELASTIC (E1)

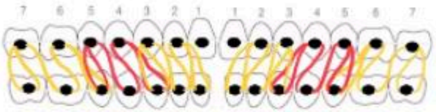

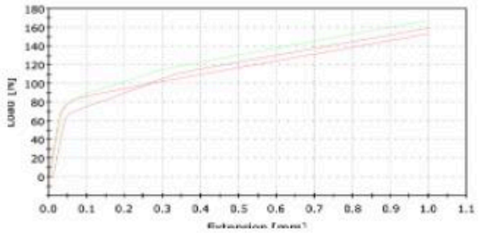

	Load (Extension 0.5 mm) [N]
1	116.95191
2	122.92224
3	130.32413
Mean	123.39943
Standard deviation	6.69887
Coefficient of variation	5.42861

INTERLOCKING ELASTIC (E2)

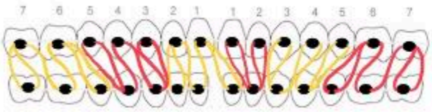

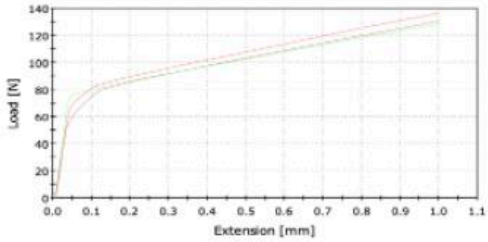

	Load (Extension 0.5 mm) [N]
1	107.72912
2	103.39453
3	102.61011
Mean	104.57792
Standard deviation	2.75706
Coefficient of variation	2.63637

OVERLAPPING BOX (E3)

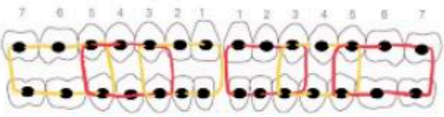

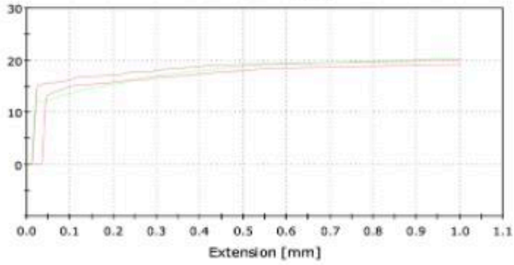

	Load (Extension 0.5 mm) [N]
1	18.00748
2	19.17148
3	18.81337
Mean	18.66411
Standard deviation	0.59618
Coefficient of variation	3.19426

NON-OVERLAPPING BOX (W1)

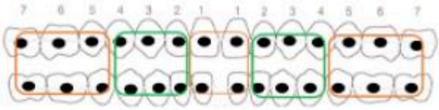

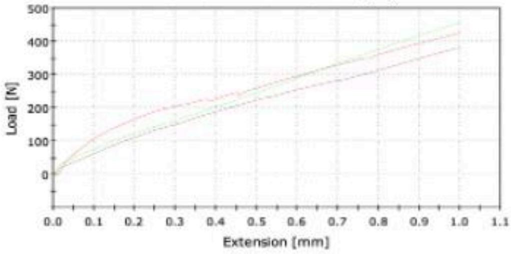

	Load (Extension 0.5 mm) [N]
1	259.55397
2	224.63609
3	245.74322
Mean	243.31109
Standard deviation	17.58554
Coefficient of variation	7.22759

OVERLAPPING BOX (W2)

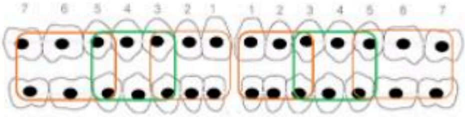

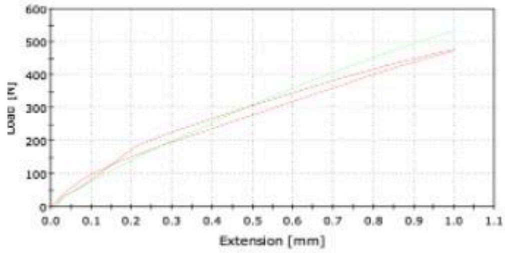

	Load (Extension 0.5 mm) [N]
1	278.59013
2	307.80114
3	310.14175
Mean	298.84434
Standard deviation	17.57966
Coefficient of variation	5.88255

ANTERIOR, POSTERIOR BOX (W3)

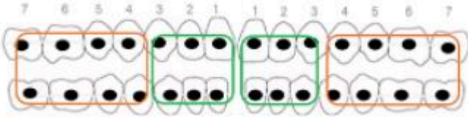

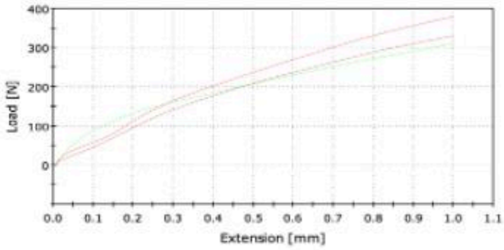

	Load (Extension 0.5 mm) [N]
1	237.24372
2	209.81289
3	208.42273
Mean	218.49311
Standard deviation	16.25337
Coefficient of variation	7.43885

Appendix A. Data collected from the pilot study
