# Supplementary material for: An in vitro study in separating tensile loads during maxillo-mandibular fixation using wire and/or elastics
Source: PLoS One. 2024 Mar 15;19(3):e0300481. doi: 10.1371/journal.pone.0300481 (PMC10942067; doi:10.1371/journal.pone.0300481)
Supplement: S1 Data — (ZIP) [file pone.0300481.s002.zip › Wire #2.is_tens.pdf]

Specimen 1 to 10

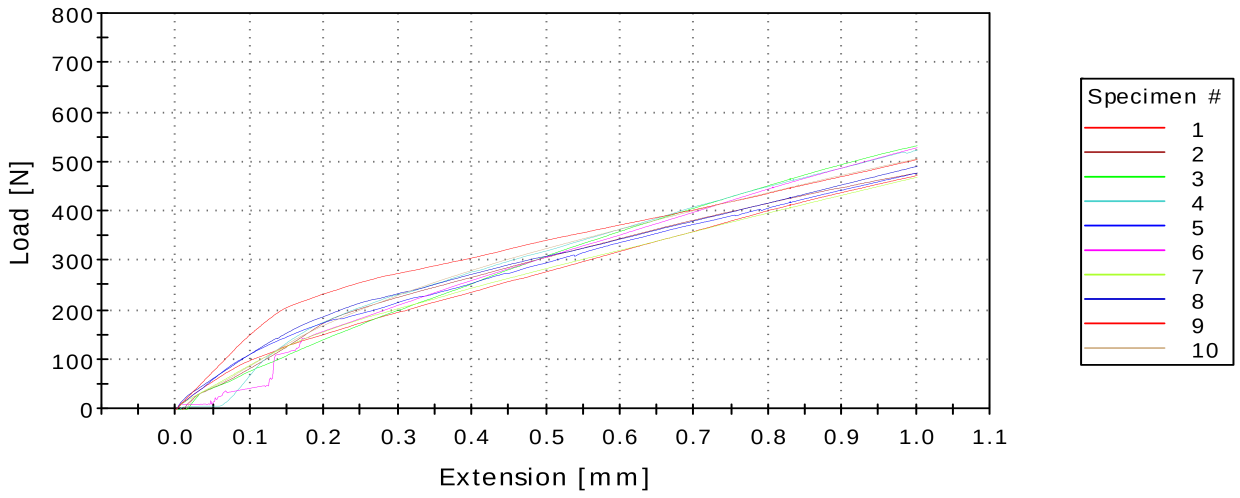

	Load [N]	extension [mm]	Rate 1 [mm/min]
1	472.30026	1.00005	0.50000
2	477.40649	0.99994	0.50000
3	532.82300	1.00005	0.50000
4	524.22803	1.00005	0.50000
5	477.84479	1.00005	0.50000
6	528.74377	1.00005	0.50000
7	468.94421	1.00005	0.50000
8	491.27231	1.00005	0.50000
9	504.81021	1.00005	0.50000
10	506.59525	1.00005	0.50000
Mean	498.49683	1.00004	0.50000
Standard deviation	24.36715	0.00004	0.00000
Coefficient of variation	4.88812	0.00359	0.00000

	Load (Extension 0.5 mm) [N]
1	278.59013
2	307.80114
3	310.14175
4	320.29186
5	296.63811
6	308.10583
7	284.95121
8	309.88453
9	342.03638
10	325.16089
Mean	308.36018
Standard deviation	18.66736
Coefficient of variation	6.05375
