# Supplementary material for: An in vitro study in separating tensile loads during maxillo-mandibular fixation using wire and/or elastics
Source: PLoS One. 2024 Mar 15;19(3):e0300481. doi: 10.1371/journal.pone.0300481 (PMC10942067; doi:10.1371/journal.pone.0300481)
Supplement: S1 Data — (ZIP) [file pone.0300481.s002.zip › Combination.is_tens.pdf]

Specimen 1 to 10

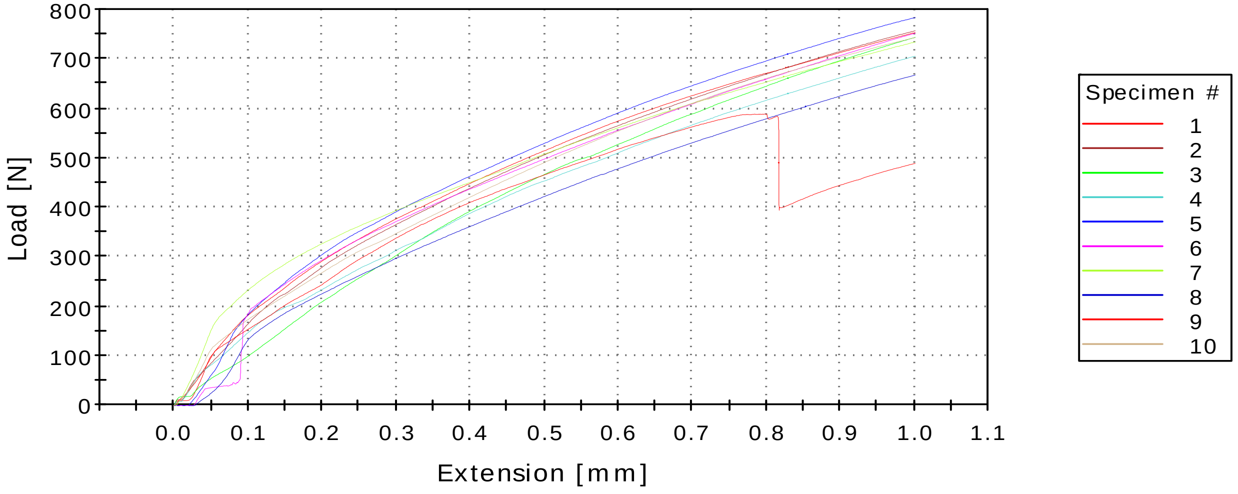

	Load [N]	extension [mm]	Rate 1 [mm/min]
1	752.80853	1.00005	0.50000
2	756.36005	1.00005	0.50000
3	742.91980	1.00005	0.50000
4	705.00555	1.00005	0.50000
5	782.86346	1.00005	0.50000
6	751.39563	1.00005	0.50000
7	733.62195	1.00005	0.50000
8	666.93616	1.00005	0.50000
9	588.61298	0.79928	0.50000
10	742.62830	1.00005	0.50000
Mean	722.31524	0.97998	0.50000
Standard deviation	56.57681	0.06349	0.00000
Coefficient of variation	7.83270	6.47863	0.00000

	Load (Extension 0.5 mm) [N]
1	514.40260
2	506.14733
3	466.78601
4	453.47733
5	529.43198
6	497.72259
7	507.46242
8	422.14467
9	465.96818
10	490.76852
Mean	> 485.43116
Standard deviation	32.69760
Coefficient of variation	6.73578
