# Supplementary material for: An in vitro study in separating tensile loads during maxillo-mandibular fixation using wire and/or elastics
Source: PLoS One. 2024 Mar 15;19(3):e0300481. doi: 10.1371/journal.pone.0300481 (PMC10942067; doi:10.1371/journal.pone.0300481)
Supplement: S1 Data — (ZIP) [file pone.0300481.s002.zip › Elastic1.is_tens.pdf]

Specimen 1 to 10

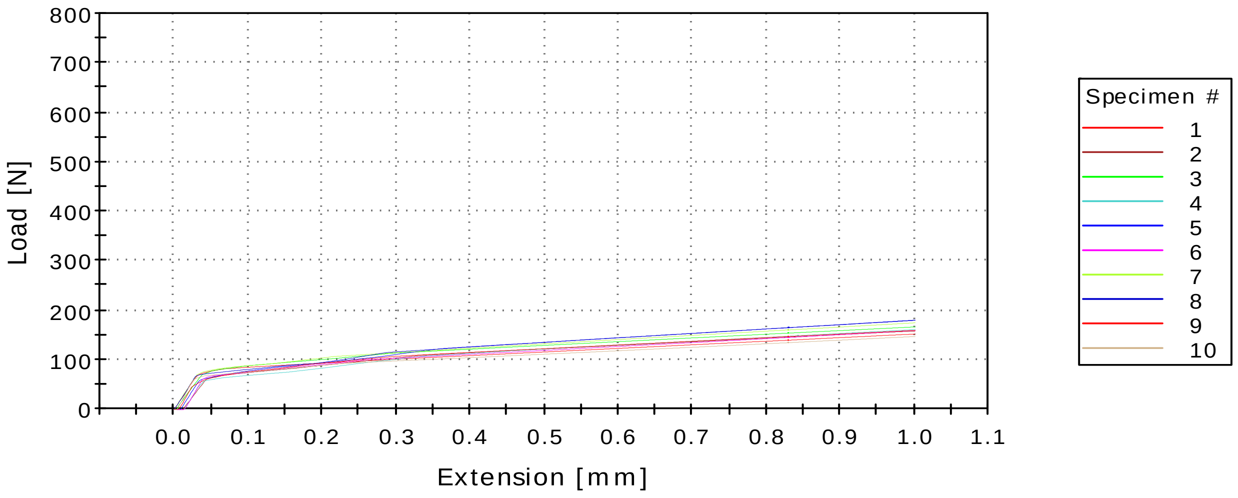

	Load [N]	extension [mm]	Rate 1 [mm/min]
1	153.15475	1.00005	0.50000
2	159.21822	1.00005	0.50000
3	167.56401	1.00005	0.50000
4	162.26672	1.00005	0.50000
5	181.30788	1.00005	0.50000
6	160.40436	1.00005	0.50000
7	175.77254	1.00005	0.50000
8	180.70934	1.00005	0.50000
9	160.44997	1.00005	0.50000
10	148.21387	1.00005	0.50000
Mean	164.90616	1.00005	0.50000
Standard deviation	11.25498	0.00000	0.00000
Coefficient of variation	6.82508	0.00000	0.00000

	Load (Extension 0.5 mm) [N]
1	116.95191
2	122.92224
3	130.32413
4	123.10621
5	136.34141
6	120.10125
7	133.37712
8	136.47841
9	123.76411
10	112.56619
Mean	125.59330
Standard deviation	8.22046
Coefficient of variation	6.54530
