# Supplementary material for: An in vitro study in separating tensile loads during maxillo-mandibular fixation using wire and/or elastics
Source: PLoS One. 2024 Mar 15;19(3):e0300481. doi: 10.1371/journal.pone.0300481 (PMC10942067; doi:10.1371/journal.pone.0300481)
Supplement: Attachment — Submitted filename: PONE-D-23-14421_R1_reviewer.pdf [file pone.0300481.s004.pdf]

PLOS ONE

An In Vitro Study in Separating Tensile Loads during Maxillo-Mandibular Fixation using Wire and/or Elastics

--Manuscript Draft--

**Manuscript Number:**	PONE-D-23-14421R1
**Article Type:**	Research Article
**Full Title:**	An In Vitro Study in Separating Tensile Loads during Maxillo-Mandibular Fixation using Wire and/or Elastics
**Short Title:**	An In Vitro Study in Separating Tensile Loads during Maxil-lo-Mandibular Fixation using Wire and/or Elastics
**Corresponding Author:**	Surakit Visuttiwattanakorn<br>Mahidol University Faculty of Dentistry<br>Bangkok, THAILAND
**Keywords:**	maxillo-mandibular fixation; intermaxillary fixation; tensile load
**Abstract:**	Intermaxillary fixation (IMF) or maxillo-mandibular fixation (MMF) is a fundamental process in stabilizing the maxilla and mandible through dental maximum intercuspation (MIP) during the management of trauma, orthognathic surgery, and reconstruction. Despite the availability of several techniques in achieving adequate maxillo-mandibular fixation, concerns have been raised regarding the sufficiency of using only latex elastics to counter displacing forces during reduction and fixation. An in vitro study was performed to determine which maxillo-mandibular fixation method between wire, elastics or a combination represents the greatest form of fixation using custom-made models stimulating dental arches. A loading rate of 1 mm/minute was used using an Instron 5566 Universal Testing Machine to separate the models unidirectionally in a vertical plane up to 1 mm apart. The tensile load was recorded at 0.5 and 1 mm vertical separation. In each sample group, each process was repeated 10 times, and an average peak tensile load was recorded. A one-way ANOVA statistical analysis at p<0.05 significance level was conducted and an outcome assessment showed statistical significance between all three subject groups. In this conducted in vitro study, a comprehensive analysis was performed to assess the ability of three distinct maxillo-mandibular fixation techniques to withstand opposing forces. The findings substantiate the superiority of the combination technique in achieving robust maxillo-mandibular fixation, positioning it as the most effective among the evaluated techniques.
**Order of Authors:**	Sudeep Pawa
	Surakit Visuttiwattanakorn
**Response to Reviewers:**	Dear Editor and Reviewers,<br><br>On behalf of all authors, we are grateful for your helpful in-depth feedback on our manuscript. We hope that you consider all raised points to be adequately addressed by our revised manuscript as described below and that the revised manuscript qualifies for publication.<br><br>Kind Regards,<br><br>Surakit Visutiwattanakorn<br><br>Editor Comments<br><br>1.Abstract: Condense the first three sentences into one to provide a concise overview. Focus more on explaining the meaning of the findings and their clinical implications.<br><br>The abstract has been revised to provide a more concise overview of the manuscript.<br><br>2.General format: Ensure that the manuscript follows the PLOS ONE format. Please

Powered by Editorial Manager® and ProduXion Manager® from Aries Systems Corporation

consult the information for authors to ensure compliance.

The manuscript has been edited to follow the PLOS ONE format as written on the "information for authors" page.

3. Introduction: Although the introduction provides a comprehensive overview of intermaxillary fixation (IMF) or maxillo-mandibular fixation (MMF), there are areas for improvement:

3.1) Clearly state the research problem or objective in a concise manner. Highlight the specific research question or knowledge gap that the study aims to address.

3.2) Establish the need for further investigation or improvement by discussing the limitations, challenges, or evidence gaps associated with current techniques. Justify the need for the study by providing relevant information from the literature.

3.3) Elaborate on the advantages and limitations of orthodontic latex elastics as an alternative to wire ligatures. Briefly discuss the advantages and disadvantages of different fixation methods to enhance the reader's understanding of the research context.

3.4) Restructure the introduction to ensure a logical flow and organization of information. Improve transitions between ideas to present a coherent and logical progression of concepts.

The introduction has been rewritten and involves all the changes recommended by the editor and reviewers. The research problem and objectives have been clearly stated so that the reader clearly understands the knowledge gap and what this study aims to address.

4.Methods: While the methods provide sufficient experimental details, include information on how the model simulates clinical situations. If there is clinical validation, provide relevant details. If not, discuss how the results can be applied to clinical patients.

The materials and methods have been reformatted to better explain how the custom-made models simulate real-life scenarios. The purpose of the study has been stated to further discuss how the results can be applied.

5.Discussion and Conclusion: Separate the Discussion and Conclusion sections. The Discussion should follow this format: 4.1) highlight important results, 4.2) compare the results with previous studies, 4.3) discuss the limitations of the study, and 4.4) indicate clinical implications and suggest future studies. Move Figure 7 from the Discussion section to the Results section.

The discussion and conclusion sections have been separated as recommended. Figure 7 has been moved and re-cited within the results section.

6. Conclusion: Provide a clear and direct summary of the results in terms of addressing the hypothesis. Since the manuscript lacks a clear clinical problem and hypothesis, ensure the conclusion is based on the obtained results.

The conclusion has been totally re-written as per the editors and reviewer's recommendation.

Journal Requirements

1.Please ensure that your manuscript meets PLOS ONE's style requirements, including those for file naming.

I have re-formatted my manuscript according the PLOS ONE's style requirements. Please let me know if any additional changes need to be made.

2.PLOS requires an ORCID iD for the corresponding author in Editorial Manager on papers submitted after December 6th, 2016. Please ensure that you have an ORCID iD and that it is validated in Editorial Manager.

I have updated my profile to include my ORCID iD in the Editorial Manager Portal.

3.We note that Figures 1, 2, 3, and 4 in your submission contain copyrighted images. All PLOS content is published under the Creative Commons Attribution License (CC BY 4.0), which means that the manuscript, images, and Supporting Information files will be freely available online, and any third party is permitted to access, download, copy, distribute, and use these materials in any way, even commercially, with proper attribution.

Figures 1-4 are original photographs taken by Sudeep Pawa during the experimentation phase of this study. Is it still necessary to have the copyright paperwork for this situation?

Reviewer #1: To begin with
- English language editing services very much needed for manuscript.
- This is a CAD based design which was utilised for this invitro study . Wat was the printing material utilised for this study????
- Please provide justifications and citations to say the model material doesn't interferes with testing results.
- Please justify your sample size
- Please add more correct citations under discussion. Total references of 13 for an original article is unacceptable.
- Wat is new in this study when compared to previously published articles on similar topic.
- Please provide relevant citations to your materials and methods section.
- Your study is on tensile load only. Wat about shear loads acting on mandible at time of Fixation. Anything included in your study ???

Reply to Reviewer # 1

We sincerely appreciate your insightful and thorough review of our manuscript. The manuscript has undergone grammatical revisions. As detailed within, the customized models were created through the utilization of Computer-Aided Design (CAD) and subsequently fabricated via Computer-Aided Manufacturing (CAM). These models were constructed utilizing Stainless Steel as the chosen material. While material selection lacks specific justification, it is worth noting that material properties did not influence this study's scope, which solely focused on analyzing Tensile Loads causing separation between the two models.
In the context of sample size, considering our status as a pilot study embarking on the pioneering examination of tensile loads in Maxillo-Mandibular Fixation, an initial pilot study was conducted. Sample size determination was carried out employing Stata/SE 15.1 for power analysis concerning one-way analysis of variance. This calculation incorporated a statistical significance level of 0.001, alongside a power value of 0.99, drawing on insights garnered from the pilot study's data.
Given the absence of prior experimental investigations into tensile loads encompassing varied patterns during maxillo-mandibular fixation, it is important to acknowledge that direct comparisons with previous research cannot be made. Furthermore, with regards to shear loads impacting the mandible, the study was intentionally designed to explore vertical (unidirectional) forces exclusively. It is acknowledged that this design choice represents a limitation within our study.

Reviewer #2: Nice preclinical project, not without its limitations as noted. Well written and documented. A couple of minor suggestions:
1. although the outcomes will not change, I would recommend indicating the parameters that were tested and to what level they were tested when completing the power analysis.
2. Not being an OMS or orthodontist and being familiar with the up to date state of MMF and arrangement of wires and elastics commonly used in MMF, it might help the reader to know what currently is the standard configuration that is taught and used in clinical practice to achieve fixation. If internal fixation is used is external fixation even

	needed? The OMS department in my current institution rarely in orthognatic cases uses external fixation once the internal fixation is placed. I would make comment of the current gold standard, and in what cases would you prefer external vs internal fixation. Additionally, I would also maybe make more comment of the consequences of inadequate fixation in the introduction.<br>3. Discuss experimental model limitations in greater detail and contrasting to what is done clinically- while MM bone fixation screws may be used (I am guessing not usually one for every tooth as used in this model), as indicated orthodontic brackets +- MMF screws are more commonly used. Why was one screw used for every tooth? What was the rationale? Why were orthodontic brackets not used if this is more commonly used in clinical practice? If repeating or extending this experimental model to study the effects of lateral forces, I would suggest integrating orthodontic brackets into the model (if you are able to find a way to bond to the custom 'dental model') to better represent what may be done clinically. This may be a point for discussion in the limitations of this preclinical model, along with discussion of any potential differences in the size of the custom fixation screw that was used in this study, to what it commonly available on the market (they seem quite large in diameter and not what may be used clinically). Granted this is a preclinical study with obvious limitations, clinical correlation and deeper discussion on model limitations is helpful for the reader as if products/screws that are used are not commercially available or the set up is not as it would be in a real situation then the results have minimal clinical meaning.<br>Other than that, nice project. I would be interested to see as you mentioned how it would fare up with lateral movements which if the patient is a bruxer may be the time when potentially higher forces are generated and this could impact the fixation.<br><br>Reply to Reviewer # 2<br><br>We sincerely appreciate your insightful and thorough review of our manuscript. The study has taken steps to enhance reader comprehension by rephrasing the study's parameters and detailing confidence levels or p-values. Regarding the established benchmark for achieving satisfactory Maxillomandibular Fixation (MMF), it's important to note that both techniques continue to be employed globally without a definitive consensus on a superior method. Concerning internal and external fixation, External Fixation (MMF) is frequently employed to facilitate precise and anatomical internal fixation. In the postoperative phase, external fixation serves to guide occlusion training in numerous instances. The introduction has now been supplemented with information on the ramifications of insufficient MMF.<br>For the purpose of this pilot study and to mitigate variables influencing outcomes, the authors opted for the use of screws instead of orthodontic brackets. This strategic choice eliminated potential factors tied to bracket bond strength, which would have been pertinent had brackets been employed. Clear delineations have been provided about the study's limitations, along with suggestions for future research based on this study's findings<br><br>Reviewer #3: The study is well designed and the manuscript is also acceptable for publication. However, it would be better if the clinical implication(s) of the in vitro findings are highlighted. The article seems to lack novelty.<br><br><br>Reply to Reviewer # 3<br><br>We sincerely appreciate your insightful and thorough review of our manuscript. Clinical implications of this in vitro study have been added. We hope that the changes are sufficient to qualify for publication.
**Additional Information:**	
**Question**	**Response**
**Financial Disclosure**<br><br>Enter a financial disclosure statement that describes the sources of funding for the work included in this submission. Review	The author(s) received no specific funding for this work.

the submission guidelines for detailed requirements. View published research articles from *PLOS ONE* for specific examples.<br><br>This statement is required for submission and **will appear in the published article** if the submission is accepted. Please make sure it is accurate.<br><br>**Unfunded studies**<br>Enter: *The author(s) received no specific funding for this work.*<br><br>**Funded studies**<br>Enter a statement with the following details:<br>• Initials of the authors who received each award<br>• Grant numbers awarded to each author<br>• The full name of each funder<br>• URL of each funder website<br>• Did the sponsors or funders play any role in the study design, data collection and analysis, decision to publish, or preparation of the manuscript?<br>• **NO** - Include this sentence at the end of your statement: *The funders had no role in study design, data collection and analysis, decision to publish, or preparation of the manuscript.*<br>• **YES** - Specify the role(s) played.<br><br>* typeset	
**Competing Interests**<br><br>Use the instructions below to enter a competing interest statement for this submission. On behalf of all authors, disclose any competing interests that could be perceived to bias this work—acknowledging all financial support and any other relevant financial or non-financial competing interests.<br><br>This statement is required for submission and **will appear in the published article** if the submission is accepted. Please make sure it is accurate and that any funding sources listed in your Funding Information later in the submission form are also	The authors have declared that no competing interests exist.

Powered by Editorial Manager® and ProduXion Manager® from Aries Systems Corporation

declared in your Financial Disclosure statement.<br><br>View published research articles from *PLOS ONE* for specific examples.<br><br>**NO authors have competing interests**<br><br>Enter: *The authors have declared that no competing interests exist.*<br><br>**Authors with competing interests**<br><br>Enter competing interest details beginning with this statement:<br><br>*I have read the journal's policy and the authors of this manuscript have the following competing interests: [insert competing interests here]*<br><br>* typeset	
**Ethics Statement**<br><br>Enter an ethics statement for this submission. This statement is required if the study involved:<br><br>• Human participants<br>• Human specimens or tissue<br>• Vertebrate animals or cephalopods<br>• Vertebrate embryos or tissues<br>• Field research<br><br>Write "N/A" if the submission does not require an ethics statement.<br><br>General guidance is provided below. Consult the submission guidelines for detailed instructions. **Make sure that all information entered here is included in the Methods section of the manuscript.**	N/A

**Format for specific study types**<br><br>**Human Subject Research (involving human participants and/or tissue)**<br>• Give the name of the institutional review board or ethics committee that approved the study<br>• Include the approval number and/or a statement indicating approval of this research<br>• Indicate the form of consent obtained (written/oral) or the reason that consent was not obtained (e.g. the data were analyzed anonymously)<br><br>**Animal Research (involving vertebrate animals, embryos or tissues)**<br>• Provide the name of the Institutional Animal Care and Use Committee (IACUC) or other relevant ethics board that reviewed the study protocol, and indicate whether they approved this research or granted a formal waiver of ethical approval<br>• Include an approval number if one was obtained<br>• If the study involved *non-human primates*, add *additional details* about animal welfare and steps taken to ameliorate suffering<br>• If anesthesia, euthanasia, or any kind of animal sacrifice is part of the study, include briefly which substances and/or methods were applied<br><br>**Field Research**<br><br>Include the following details if this study involves the collection of plant, animal, or other materials from a natural setting:<br>• Field permit number<br>• Name of the institution or relevant body that granted permission	
**Data Availability**<br><br>Authors are required to make all data underlying the findings described fully available, without restriction, and from the time of publication. PLOS allows rare exceptions to address legal and ethical concerns. See the PLOS Data Policy and FAQ for detailed information.	Yes - all data are fully available without restriction

A Data Availability Statement describing where the data can be found is required at submission. Your answers to this question constitute the Data Availability Statement and **will be published in the article**, if accepted.<br><br>**Important:** Stating 'data available on request from the author' is not sufficient. If your data are only available upon request, select 'No' for the first question and explain your exceptional situation in the text box.<br><br>Do the authors confirm that all data underlying the findings described in their manuscript are fully available without restriction?	
**Describe where the data may be found in full sentences. If you are copying our sample text, replace any instances of XXX with the appropriate details.**<br><br>• If the data are **held or will be held in a public repository**, include URLs, accession numbers or DOIs. If this information will only be available after acceptance, indicate this by ticking the box below. For example: *All XXX files are available from the XXX database (accession number(s) XXX, XXX.)*.<br>• If the data are all contained **within the manuscript and/or Supporting Information files**, enter the following: *All relevant data are within the manuscript and its Supporting Information files.*<br>• If neither of these applies but you are able to provide **details of access elsewhere**, with or without limitations, please do so. For example:<br><br>*Data cannot be shared publicly because of [XXX]. Data are available from the XXX Institutional Data Access / Ethics Committee (contact via XXX) for researchers who meet the criteria for access to confidential data.*<br><br>*The data underlying the results presented in the study are available from (include the name of the third party*	All relevant data are within the manuscript and its Supporting Information files.

and contact information or URL).<br>• This text is appropriate if the data are owned by a third party and authors do not have permission to share the data.<br><br>* typeset	
Additional data availability information:	

Powered by Editorial Manager® and ProduXion Manager® from Aries Systems Corporation

Manuscript Click here to access/download;Manuscript;Manuscript without Tracking.docx

Experimental Study

An In Vitro Study in Separating Tensile Loads during Maxillo-Mandibular Fixation using Wire and/or Elastics

Sudeep Pawa [1¶], Surakit Visuttiwattanakorn [1¶*]

[1] Department of Oral and Maxillofacial Surgery, Faculty of Dentistry, Mahidol University, Bangkok, Thailand

* Corresponding author

Email: surakit_vis@mahidol.ac.th

Abstract. Intermaxillary fixation (IMF) or maxillo-mandibular fixation (MMF) is a fundamental process in stabilizing the maxilla and mandible through dental maximum intercuspation (MIP) during the management of trauma, orthognathic surgery, and reconstruction. Despite the availability of several techniques in achieving adequate maxillo-mandibular fixation, concerns have been raised regarding the sufficiency of using only latex elastics to counter displacing forces during reduction and fixation. An in vitro study was performed to determine which maxillo-mandibular fixation method between wire, elastics or a combination represents the greatest form of fixation using custom-made models stimulating dental arches. A loading rate of 1 mm/minute was used using an Instron 5566 Universal Testing Machine to separate the models unidirectionally in a vertical plane up to 1 mm apart. The tensile load was recorded at 0.5 and 1 mm vertical separation. In each sample group, each process was repeated 10 times, and an average peak tensile load was recorded. A one-way ANOVA statistical analysis at $p<0.05$ significance level was conducted and an outcome assessment showed statistical significance between all three subject groups. In this conducted in vitro study, a comprehensive analysis was performed to assess the ability of three distinct maxillo-mandibular fixation techniques to withstand opposing forces. The findings substantiate the superiority of the combination technique in achieving robust maxillo-mandibular fixation, positioning it as the most effective among the evaluated techniques.

Keywords: maxillo-mandibular fixation, intermaxillary fixation, tensile load

1. Introduction

Intermaxillary fixation (IMF) or maxillo-mandibular fixation (MMF) is a crucial technique that aims for the stabilization of the maxilla and mandible through the achievement of dental maximum intercuspation, particularly in cases of facial trauma, reconstruction, and orthognathic surgery [1]. For example, during facial fractures involving the maxilla or mandible, maxillo-mandibular fixation (MMF) is used to adequately reduce fractured segments prior to internal fixation with plates and screws. Similarly, during orthognathic surgery, surgeons apply maxillo-mandibular fixation at the intermediate stage of bimaxillary surgery or before applying internal fixation [2,3]. Historically, traditional methods for achieving maxillo-mandibular fixation in dentate patients encompassed techniques like Erich's arch bars or interdental wiring [3,4]. The most recent introduction of intermaxillary fixation screws in 1989 represented a notable advancement in this field [3,4]. Maxillomandibular screws and orthodontic brackets with surgical hooks have become the two most commonly used techniques in orthognathic surgery [1,5,6]. In the past, maxillo-mandibular fixation was performed using wire ligatures, usually achieved with 26 or 24-gauge pre-stretched stainless-steel wires which often caused discomfort, pain, or soft tissue damage for the patients during the immobilization period [1]. However, due to concerns about the potential source of needlestick injuries in the contaminated environment of the oral cavity which represents a health risk for surgeons and assistants, it is no longer considered the gold standard [2].

In response, orthodontic latex elastics are used as an alternative to wire ligatures [7]. A recent study showed that heavy orthodontic elastics had a force decay of 31% after 24 hours in simulated oral environments [6]. Statistical analysis showed statistical significance in force reduction in the first 24 hours however, force decay between 1-14 days was not significant [6]. Evidence suggests elastics offer adequate traction even in scenarios where the use of wires may be limited [2,6,7,8]. The goal of maxillo-mandibular fixation is to resist displacing forces whilst the

new position of the alveolus or bony segments is established and fixed with internal fixation [2,9]. Despite the importance of these techniques, a significant research gap exists. Inadequate intermaxillary fixation (IMF) following oral and maxillofacial procedures can lead to malocclusion, delayed healing, bone nonunion or malunion, infections, nerve damage, aesthetic issues, functional difficulties, and chronic pain. Proper alignment and immobilization are crucial to prevent these complications and ensure successful recovery and outcomes. Although, multiple methods for achieving optimal maxillo-mandibular fixation have been proposed and used, no prior lab or clinical investigation has comprehensively examined the tensile loads applied during the application of separating forces in various maxillo-mandibular fixation techniques. The primary objective of this study is to determine which maxillo-mandibular fixation method between wire, elastics, and combination represents the most effective form of maxillo-mandibular fixation method. By providing numerical evidence of distinct tensile loads associated with different maxillo-mandibular fixation techniques, this study aims to address this critical knowledge gap and contribute to the advancement of clinical practices in this domain.

2. Materials and Methods

Two models as depicted in Fig 1 were designed and fabricated using Siemens NX CAD using computer-aided design and manufacturing with stainless steel. The models were meticulously designed and fabricated to closely replicate both the maxillary and mandibular arches, consisting of seven screws representing seven teeth in each quadrant. Custom-milled screws as illustrated in Fig 2 were fabricated using a wire cut technique to mimic maxillo-mandibular screws for the fixation of ligature wire (Stainless steel 0.020” Gauge 24, Lot No. 14662939, Fort Wayne Metals, Indiana, USA) and orthodontic latex elastics (Ram Zoo Pak Elastics, 6 Oz/170g, ¼”, 6.35 mm, Lot No. 082091864, Ormco Corporation, California, USA). This experimental study allowed for a detailed investigation

into the mechanics and outcomes of various fixation methods, shedding light on the performance characteristics of wire ligatures and orthodontic latex elastics in the context of maxillo-mandibular fixation.

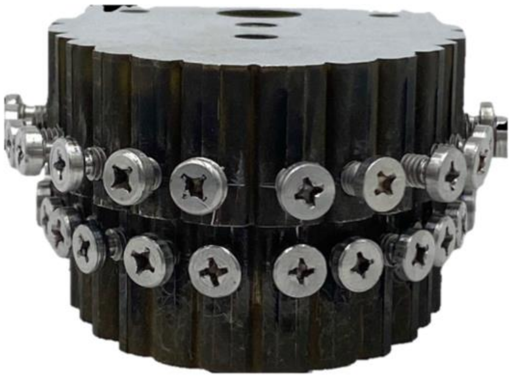

The opening of the upper and lower models does not represent the physiologic opening of the human jaws. The human jaws are related to each other via the TMJ. This allows the jaws to open along a curved path for the initial 25mm.

The difference between the opening pathway (which is along a curve) of human jaws and the seperation pathway of these models (which appears to be almost vertical) makes the experimental results invalid for clinical interpretation.

Fig 1. Custom-made dental models through CAD/CAM technique

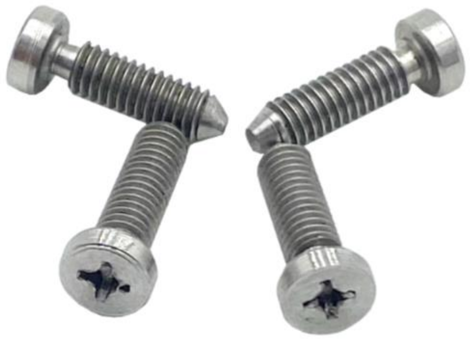

Fig 2. Screws designed and fabricated to represent IMF screws and orthodontic brackets

In order to ascertain the optimal fixation technique, a preliminary pilot study was carried out. This preliminary investigation aimed to identify the most stable fixation pattern among various fixation patterns, as depicted in Appendix A (Supplemental File). Upon analyzing the outcomes of the pilot study, the interlocking with an overlapping elastic group (E1) and the overlapping box wire group (W2) showed the highest ability to resist displacing forces

when tensile loads were applied and were selected as the representative sample groups representing wire and elastic techniques in this study.

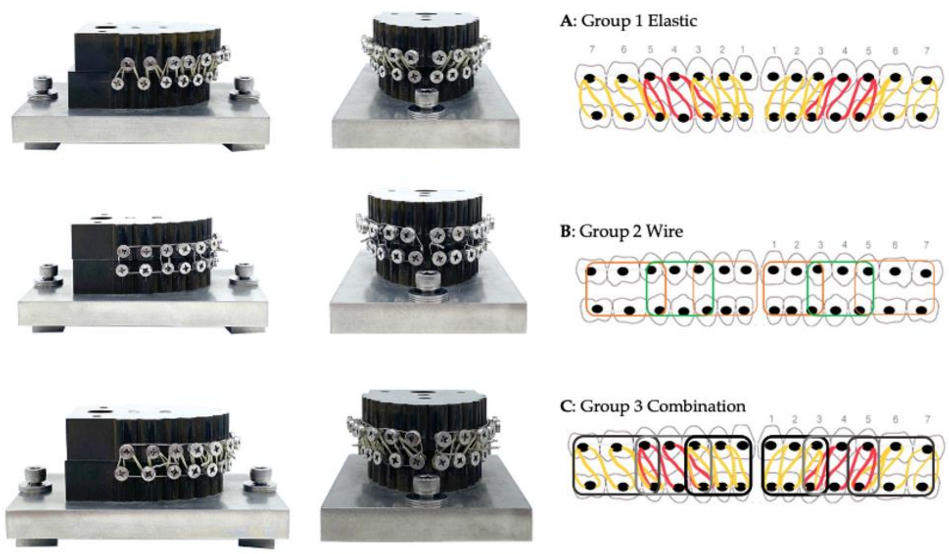

Fig 3 (A, B, C). Different MMF techniques representing all 3 sample groups

The study’s design comprised of three sample groups consisting of wire (Fig 3B), elastic (Fig 3A), and a combination group (Fig 3C); which combined the fixation pattern of both the wire and elastic groups. Sample size calculation using Stata/SE 15.1 for power analysis for one-way analysis of variance with statistical significance set at 0.001 and a power of 0.99 was calculated using data from the pilot study. According to the calculation, a minimum sample group of 4 was established. However, for this particular study, a larger sample group of 10 was employed. The samples were tested using a 1000 Newton load cell using the Instron 5566 Universal Testing Machine (Instron Ltd., Buckinghamshire, England) (DT-40-73). Maxillomandibular fixation was executed in each sample according to the specified sample group parameters and loaded on and tested using the universal testing machine until 0.5

mm and 1 mm of vertical separation between the upper and lower models was achieved as demonstrated in Fig 4A, 4B, 4C. Each process was repeated 10 times and the average peak tensile load was recorded and plotted.

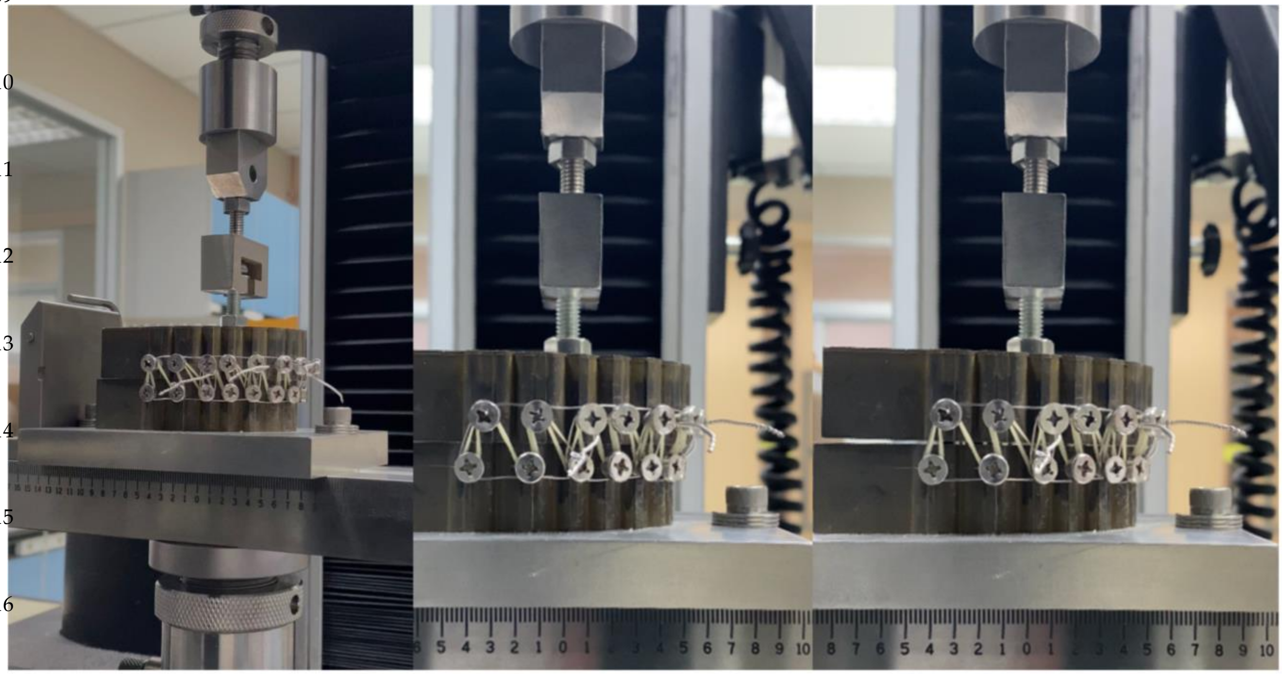

Fig 4A **Fig 4B** **Fig 4C**

Fig 4A. Orientation of dental models on the Instron 5566 Universal Testing Machine
Fig 4B. Models at rest position at 0 mm separation
Fig 3C. Models at complete 1 mm separation (complete cycle)

Descriptive statistics was calculated using Microsoft Excel and SPSS (Statistical Package for the Social Sciences) version 25 (IBM, Chicago, IL, USA). Data was analyzed using a one-way ANOVA and multiple comparisons between each group was evaluated for statistical significance using Bonferroni's Test. The level of significance was set at p<0.05. A comparison between all 3 groups is shown in Table 1.

Table 1 Comparing tensile loads using elastic, wire, and combination methods

	Elastic	Wire	Combination	P-value
Load (N): 1 mm	164.91±11.25	498.50±24.37	741.91±34.90	<0.001*
Load (N): 0.5 mm	125.59±8.22	308.36±18.67	487.45±31.98	<0.001*

Data were expressed as mean±SD and analyzed with One-way ANOVA

*Statistically significant at the 0.05 level

3. Results

This study showed that the average tensile load to achieve 1 mm of vertical separation of the models in the elastic group was at 164.91±11.25 N, the wire group at 498.50±24.37 N, and the combination group at 741.91±34.90 N (Fig 5). Statistical analyses using one-way ANOVA showed the average tensile load difference between all 3 groups was statistically significant at the 0.05 level (p<0.001) (Table 1, Fig 5).

Multiple comparisons was therefore performed using Bonferroni's test to evaluate the statistical difference among the 3 groups. The mean difference in tensile load to resist 1 mm separation between the wire and elastic groups was statistically significant at the 0.05 level (p<.001). A mean difference in tensile load was 333.59 N (95%Cl 304.57, 362.61), exhibiting that the wire group had an average tensile load 3.02 times greater than that of the elastic group. When comparing the combination group to the elastic group, the statistical analysis also showed significance at the 0.05 level (p<.001). A mean difference in tensile load was at 576.99 N (95%Cl 547.98, 606.02) or 4.50 times greater than the elastic group (Fig 5). Lastly, when comparing the combination group to the wire group, statistical significance was also reported showing a mean difference in tensile load at 243.91 N (95%Cl 214.39, 272.43), showing the combination group had an average tensile load to be 1.49 times greater than the average load of the wire group (Table 2, Fig 5).

Table 2 Multiple comparison between sample groups

Group vs. Group	Mean difference (95%CI)	Standard error	P-value
**Load (N): Extension 1 mm**			
Wire vs. Elastic	333.59 (304.57, 362.61)	11.37	<0.001*
Combination vs. Elastic	576.99 (547.98, 606.02)	11.37	<0.001*
Combination vs. Wire	243.41 (214.39, 272.43)	11.37	<0.001*
**Load: Extension 0.5 mm (N)**			
Wire vs. Elastic	182.77 (157.77, 207.76)	9.79	<0.001*
Combination vs. Elastic	361.85 (336.86, 386.85)	9.79	<0.001*
Combination vs. Wire	179.09 (154.09, 204.08)	9.79	<0.001*

Multiple comparisons were analyzed with Bonferroni method

* The mean difference is significant at the 0.05 level

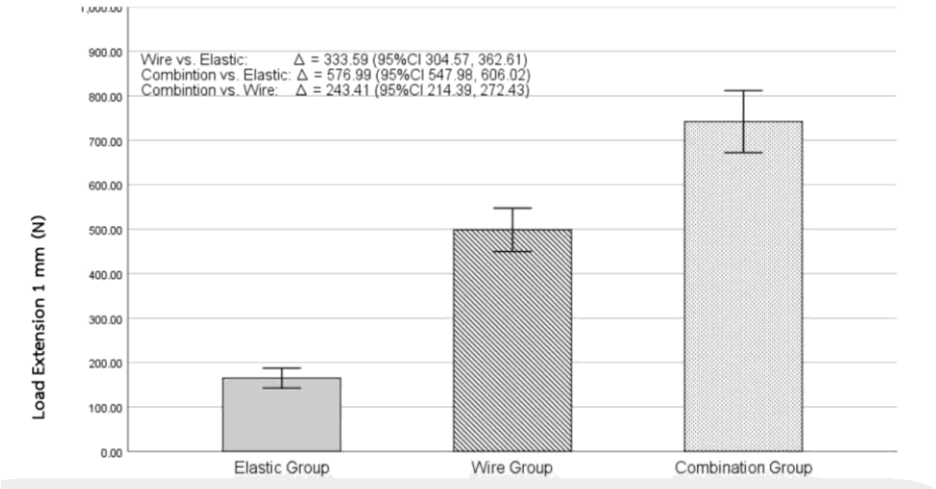

Fig 5. Mean tensile loads of maxillo-mandibular fixation between all 3 groups at 1 mm separation

Statistical analyses of the same subject groups were also performed evaluating the separation of the dental models at 0.5 mm using the same study parameters. The average tensile load in the elastic group was 125.59±8.22 N, the wire group at 308.36±18.67 N, and the combination group at 487.45±31.98 N, respectively (Fig 6). Statistical analysis using one-way ANOVA showed the average tensile load difference between all 3 groups was statistically significant at 0.05 ($p<0.001$) (Table 1). Multiple comparisons were therefore performed using Bonferroni's test to

evaluate the statistical difference between all 3 groups. The mean difference in tensile load to resist 0.5 mm separation between the wire group compared to the elastic was statistically significant at the 0.05 level ($p<0.001$). A mean difference in tensile load was 182.77 N (95%Cl 157.77, 207.76), displaying that the wire group had an average tensile load 2.46 times greater than that of the elastic group. When comparing the combination group to the elastic group, the statistical analysis also showed significance at the 0.05 level ($p<0.001$). A mean difference in tensile load was at 361.85 N (95%Cl 336.86, 386.85) or the combination group could resist displacing forces 3.88 times that of the elastic group. Lastly, when comparing the combination group to the wire group, statistical significance was also reported showing a mean difference in tensile load at 179.09 N (95%Cl 154.09, 204.08), meaning the combination group had an average tensile load to be 1.58 times the average load of the wire group (Table 2, Fig 6). The data from this study was therefore plotted as shown in Fig 7, showing a line graph that demonstrates the differences in tensile loads between all 3 sample groups at 0.5 and 1 mm vertical separation.

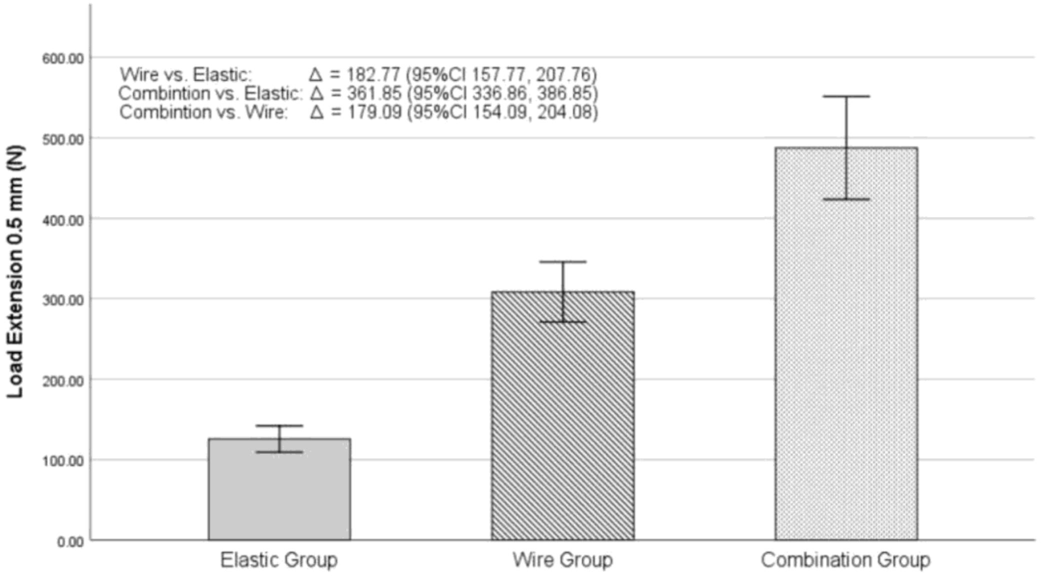

Fig 6. Mean tensile loads of maxillo-mandibular fixation between at 3 groups at 0.5 mm separation

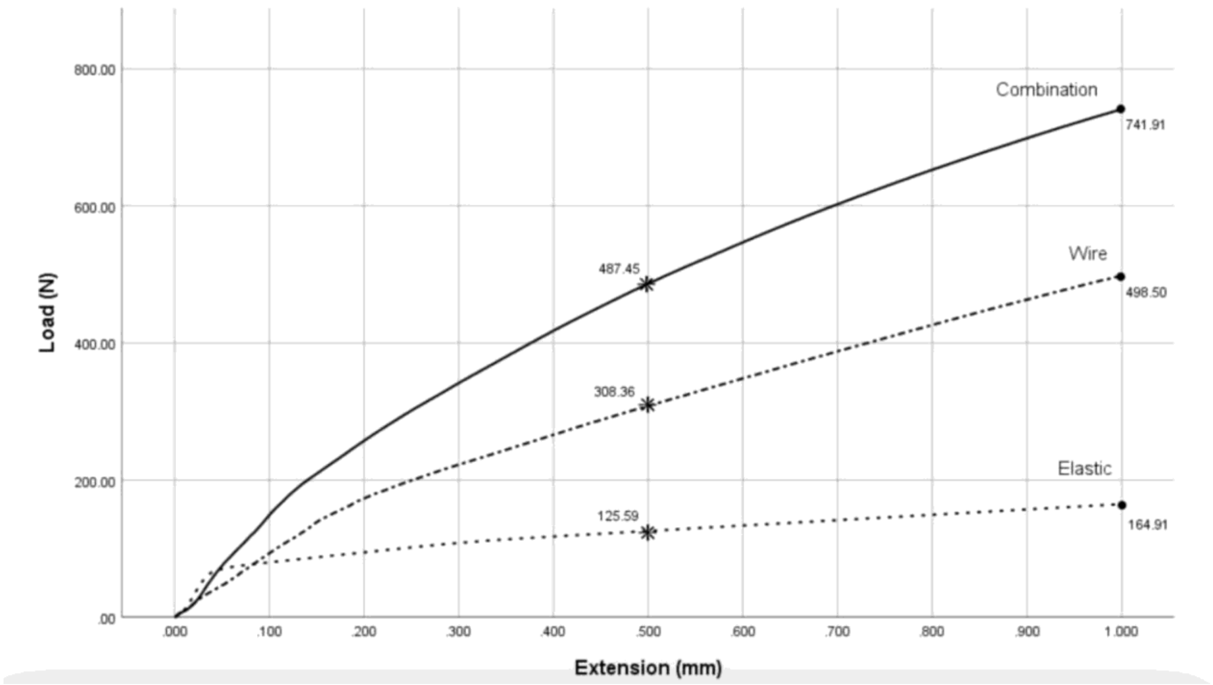

Fig 7. Line graph showing the average tensile loads at both 0.5 and 1 mm separation

4. Discussion

In this conducted in vitro study, a comprehensive analysis was performed to assess the ability of three distinct maxillo-mandibular fixation techniques to withstand opposing forces. These techniques comprised of the elastic group, the wire group, and the combination group. Statistical significance was observed in this comparison, yielding valuable insights into their relative efficacy. Data shows that the best fixation technique was the combination group followed by the wire group and lastly the elastic group, respectively. When analyzing the average tensile loads that each of these groups could endure before leading to a separation of at least 0.5 mm between the dental models, the elastic group emerged as the least resistant to opposing forces. The data further highlights a substantial discrepancy between the group with highest force-resisting capability (the combination group) and the group with the lowest (the elastic group). This disparity was significant, revealing a striking 400% difference in their respective

abilities to withstand opposing forces. These findings substantiate the superiority of the combination technique in achieving robust maxillo-mandibular fixation, positioning it as the most effective among the evaluated techniques.

In 1993, A.T. Smith highlighted the potential risk of needlestick injuries associated with traditional wiring in contaminated environments, posing health hazards to healthcare providers. A systematic review by Bouya et al. in 2020 demonstrated the alarming global prevalence of needlestick injuries, some of which were attributed to intraoperative wire-related incidents, affecting as many as 2 million healthcare workers annually. While elastics could mitigate the needlestick risk, our in vitro study data establishes that relying solely on elastics for maxillo-mandibular fixation is insufficient in resisting opposing forces when compared to the wire and combination techniques.

A study in 1970 by Yildirim studied the maximum opening and closing forces exerted by different skeletal types. The sample groups consisted of skeletal open and closed bites. The study found that there was no statistical difference between opening forces between the two groups. The average opening forces measured in the study were equivalent to 137-169 N [11]. Whereas a study by Brunton et al. in 2018 with 149 patients aged 20-60 years with overall good and general health found that in a large sample of participants of a broad age range and a demographically diverse background, jaw opening forces were greater in males than females. Men had greater maximum opening forces with median values of 157.77 N compared to women at 166.61 N [12]. Our in vitro study data sheds light on maxillo-mandibular fixation using elastics alone. It demonstrates that elastics can only resist tensile loads causing a 0.5 mm separation of dental models at 126 N and a 1 mm separation at 165 N which is insufficient to resist the maximum opening forces in men and women as studied by Yilridum and Brunton et al. Transitioning to clinical scenarios, we reviewed the maximum push force that can be exerted through human force, particularly relevant during the surgical phase when applying temporary maxillo-mandibular fixation application or fracture reduction phases. Das et al. in 2004 examined isometric pull and push strength profiles in both genders

and across different positions. Notably, the study found that maximum push strength in males ranged from 140 N to 227 N, depending on the seated or standing position. In females, the values ranged from 96 N to 140 N, again based on seating and standing positions [13].

This comprehensive examination of prior research and our in vitro findings collectively underscores the intricate interplay between technique efficacy, anatomical factors, and the practical forces encountered in clinical scenarios, providing a nuanced perspective on maxillo-mandibular fixation and its potential clinical implications.

5. Conclusion

From this study, it can be established that maxillomandibular fixation using the combination technique could resist forces at least 1.5x or 150% more when compared to the wire group and at least 4x or 400% more when compared to the elastic group as shown in Fig 7.

Furthermore, a conclusion can be made after reviewing the maximum opening forces exerted by human force and maximum push force exerted in both a standing and sitting position that using elastic alone for maxillomandibular fixation cannot achieve adequate stability sufficient in opposing displacing forces during reduction and fixation. As this study is a pilot study and the first of its kind to study the tensile load from maxillo-mandibular fixation, additional studies may be needed before a clinical recommendation can be made. This study, conducted in a controlled in vitro environment utilizing the Instron 5566 Universal Testing Machine to vertically displace dental models, provides an initial understanding of tensile loads. However, it’s vital to acknowledge that in actual anatomical scenarios, opposing forces between the mandible and maxilla encompass multidirectional dimensions, not merely vertical and horizontal. In light of these considerations, it is prudent to interpret the numerical findings from this study with a view toward more comprehensive anatomical evaluations. Alternatively, a clinical study might be envisaged to validate the clinical implications of the study’s findings. In the future, investigations should delve into

the tensile strength exhibited when models are displaced in multiple directions, aiming to provide a more comprehensive and robust understanding of maxillo-mandibular fixation mechanics in complex clinical settings.

Acknowledgments: The authors could like to acknowledge Mr. Thammasak Vimonkiattikun for his expertise and assistance in the designing and milling process of the customized dental models and screws.

Author Contributions: Conceptualization, S.P. and S.V.; methodology, S.P. and S.V.; validation, S.P. and S.V.; formal analysis, S.P. and S.V.; investigation S.P., data curation, S.P.; writing- original draft preparation S.P.; writing-reviewing and edition, S.P. and S.V. All authors have read and agreed to the published version of the manuscript.

Funding: This research received no external funding.

Institutional Review Board Statement: Not applicable.

Informed Consent Statement: Not applicable.

Conflicts of Interest: The authors declare no conflict of interest.

References

1. Cornelius C-P, Ehrenfeld M. The use of MMF screws: Surgical technique, indications, contraindications, and common problems in review of the literature. Craniomaxillofacial Trauma & Reconstruction. 2010;3(2):55–80. doi:10.1055/s0030-1254376
2. Smith AT. The use of orthodontic chain elastic for temporary intermaxillary fixation. British Journal of Oral and Maxillofacial Surgery. 1993;31(4):250 1.doi:10.1016/0266-4356(93)90150-u
3. Manjrekar S, Joshi A. Use of mandibulo-maxillary fixation screws for the treatment of maxillofacial fractures. Dentistry. 2017;7(12). doi:10.4172/2161-1122.1000467
4. Qureshi A, Reddy U, Warad N, Badal S, Jamadar A, Qurishi N. Intermaxillary fixation screws versus Erich arch bars in mandibular fractures: A comparative study and review of literature. Annals of Maxillofacial Surgery. 2016;6(1):25.doi:10.4103/2231-0746.186129
5. Minami R, Morill L, Weber J. Intermaxillary fixation with orthodontic brackets bonded to teeth. Plastic and Reconstructive Surgery. 1974;54(4):492–4. doi:10.1097/00006534-197410000-00022
6. Khajehahmadi S, Rahpeyma A. Force relaxation of 3/16 inch heavy orthodontic latex elastics used in maxillofacial trauma in simulated jaw fracture situation. Dental Hypotheses. 2014;5(4):146. doi:10.4103/2155-8213.140592
7. Kanitkar R, Kumbhare D. Role of orthodontic elastics in the oral & maxillofacial surgical procedures. Journal of Advanced Medical and Dental Sciences Research. 2020;8(2). doi:10.21276/jamdsr

8. Hernández-Alfaro F, Rosemberg V, Masià-Gridilla J, Valls-Ontañón A. Improving accuracy of the intermediate splint in substantial intermaxillary sagittal discrepancies using an extra anterior anchorage point: Technical note. Medicina Oral Patología Oral y Cirugia Bucal. 2021; doi:10.4317/medoral.24089
9. Perez DE, Liddell A. Controversies in orthognathic surgery. Oral and Maxillofacial Surgery Clinics of North America. 2017;29(4):425–40.doi:10.1016/j.coms.2017.07.008
10. Bouya S, Balouchi A, Rafiemanesh H, Amirshahi M, Dastres M, Moghadam MP, et al. Global prevalence and device related causes of needle stick injuries among health care workers: A systematic review and meta-analysis. Annals of Global Health. 2020;86(1):35. doi:10.5334/aogh.2698
11. Erkut Yilridum, John P. Devincenzo; Maximum Opening and Closing Forces Exerted by Diverse Skeletal Types. *Angle Orthod* 1 July 1971; 41 (3): 230–235. doi: https://doi.org/10.1043/0003-3219(1971)041<0230:MOACFE>2.0.CO;2
12. Brunton PA, Loch C, Waddell JN, Bodansky HJ, Hall R, Gray A. Estimation of jaw-opening forces in adults. Orthodontics & Craniofacial Research. 2017;21(1):57–62. doi:10.1111/ocr.12215
13. Das B, Wang Y. Isometric pull-push strengths in workspace: 1. Strength Profiles. International Journal of Occupational Safety and Ergonomics. 2004;10(1):43–58. doi:10.1080/10803548.2004.11076594

Click here to access/download;Figure;Fig 1.tif

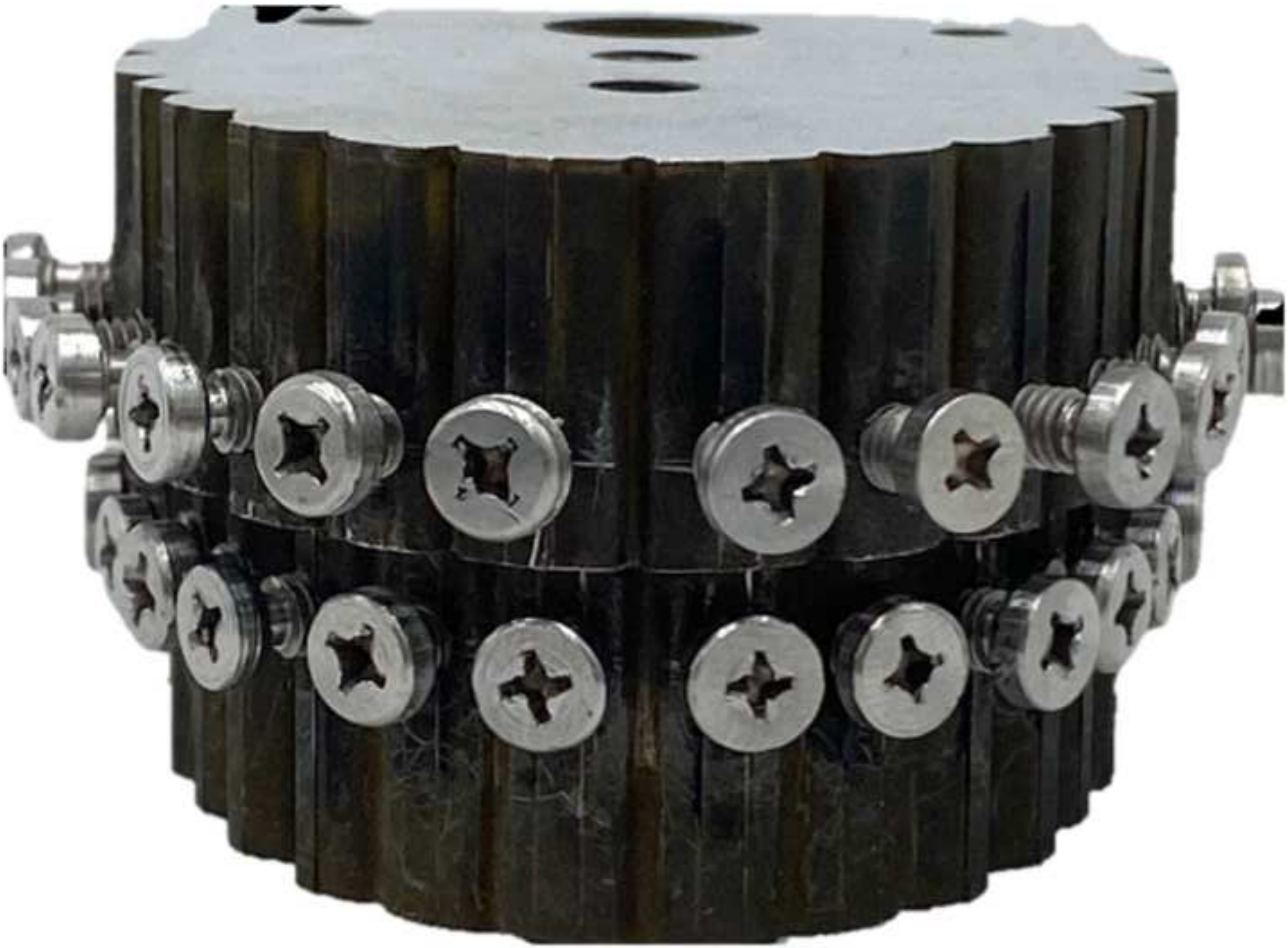

Click here to access/download;Figure;Fig 2.tif

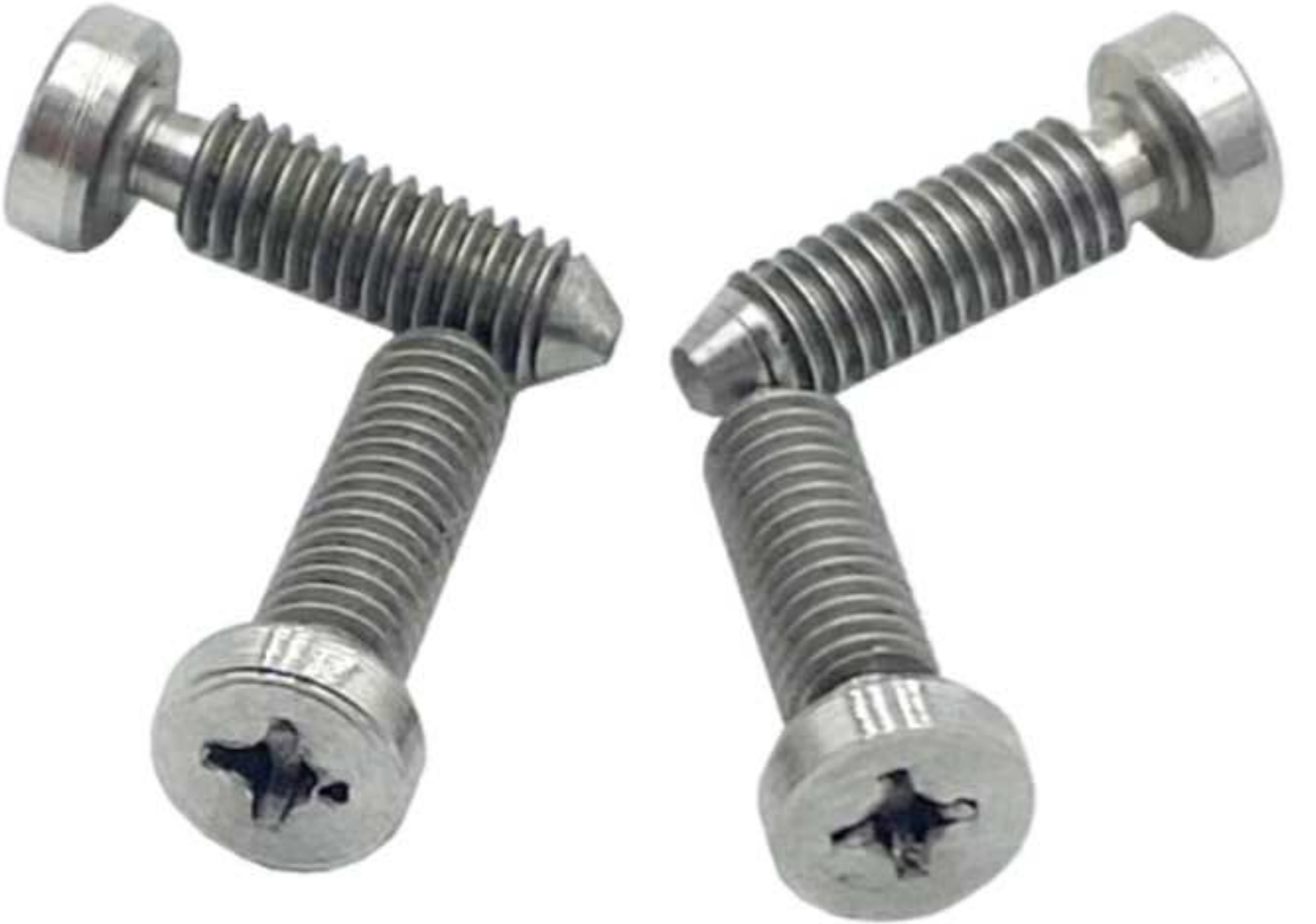

Click here to access/download;Figure;Fig 3.tif

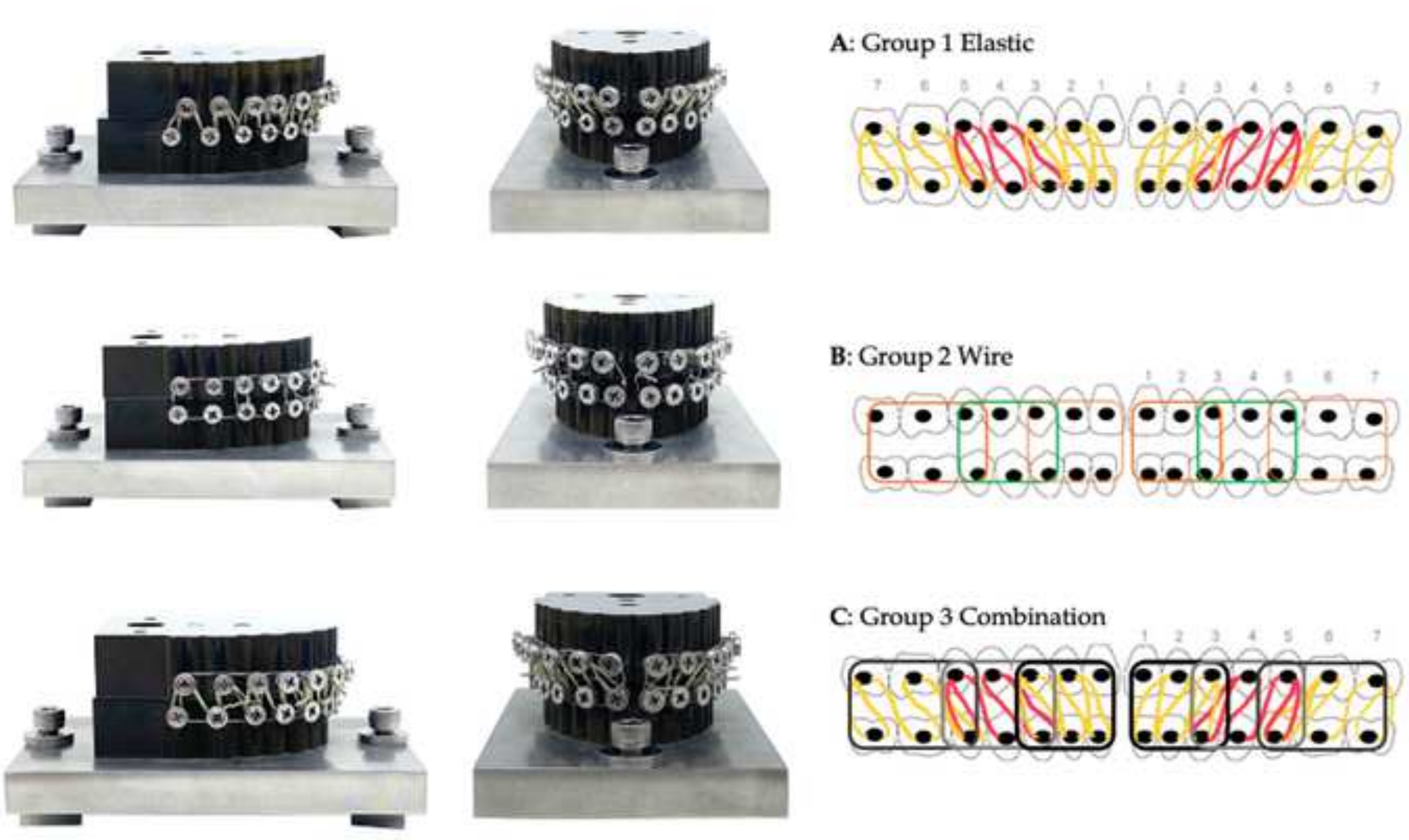

Click here to access/download;Figure;Fig 4.tif

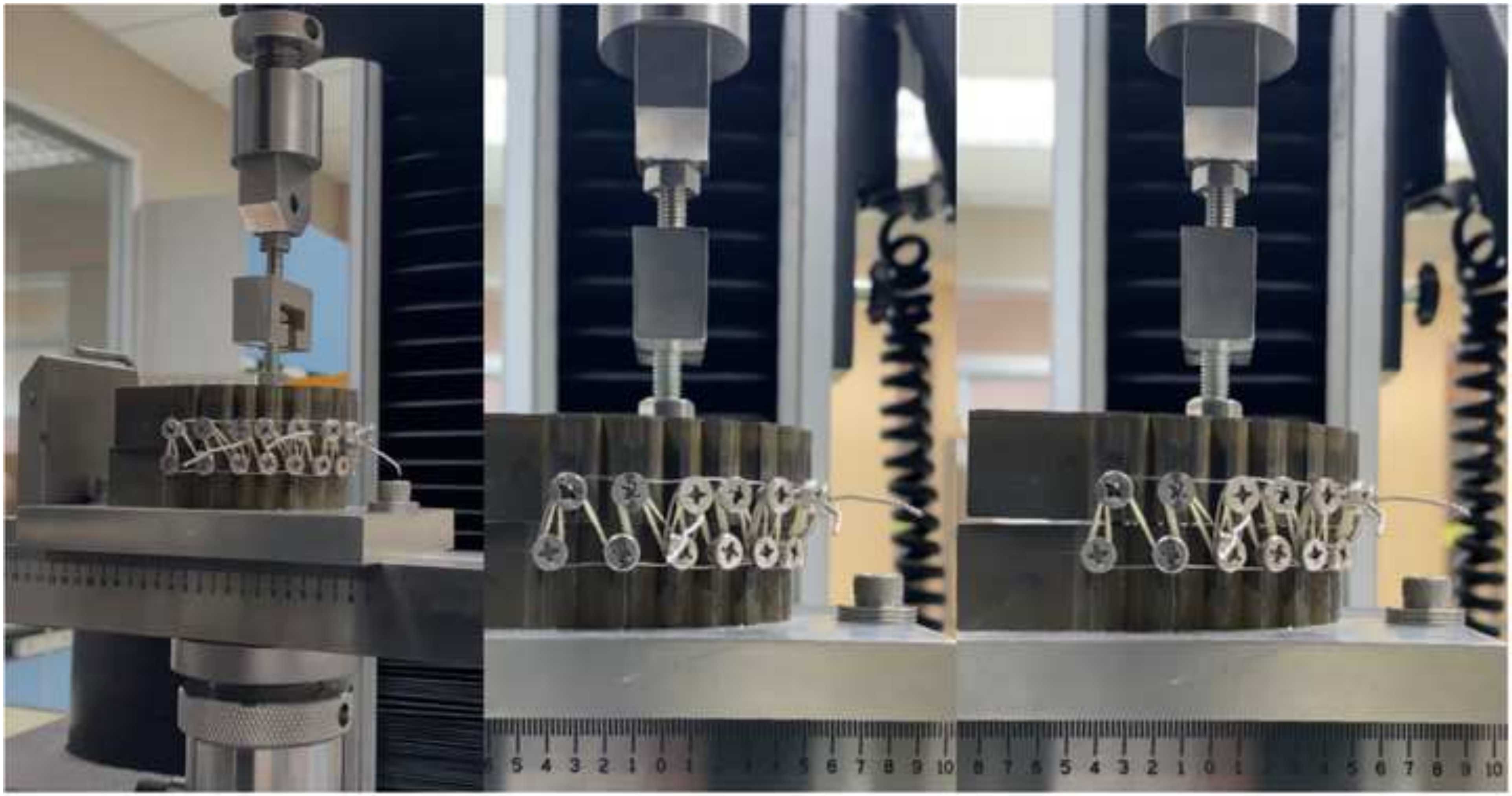

Click here to access/download;Figure;Fig 5.tif

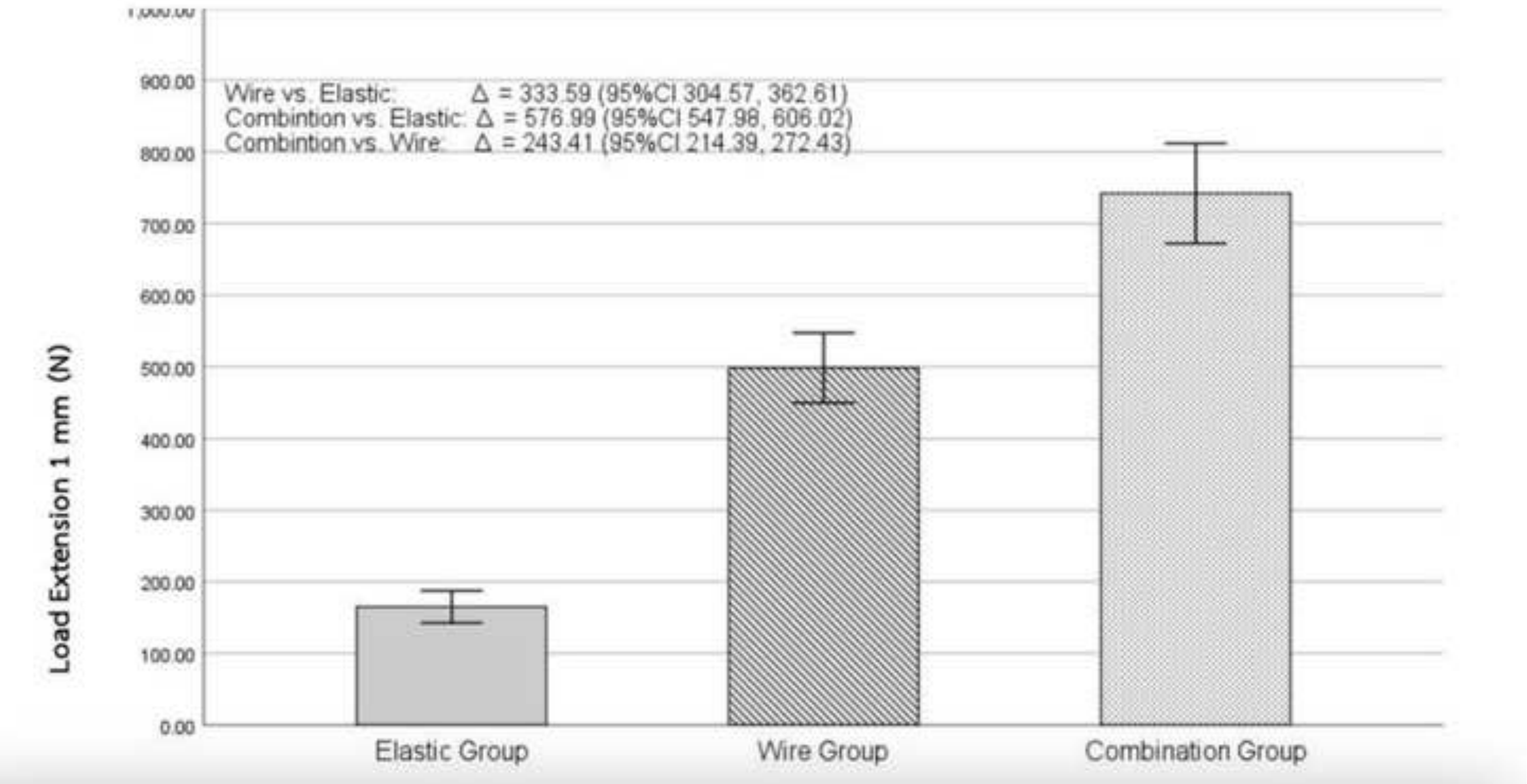

Click here to access/download;Figure;Fig 6.tif

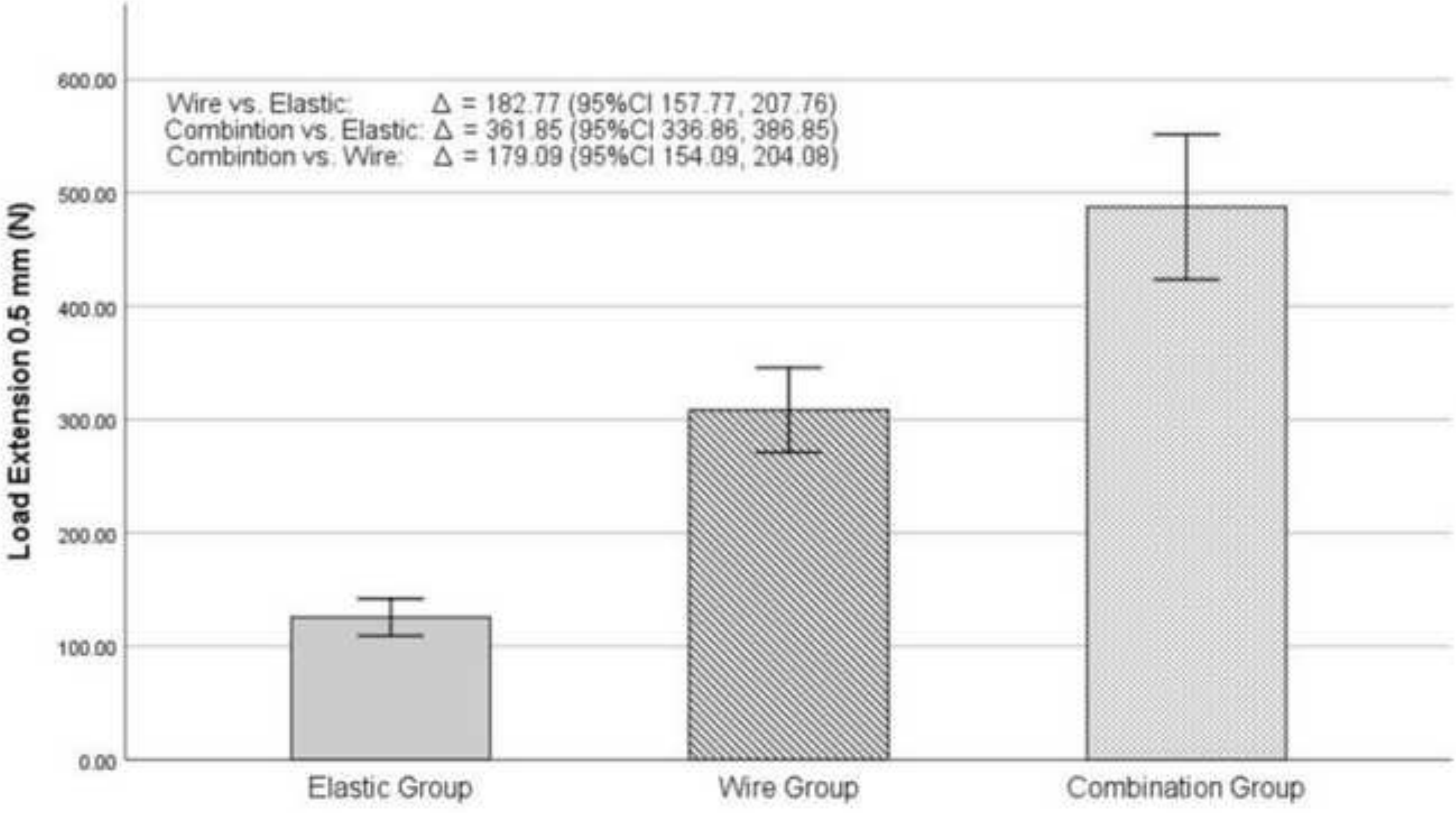

Click here to access/download;Figure;Fig 7.tif

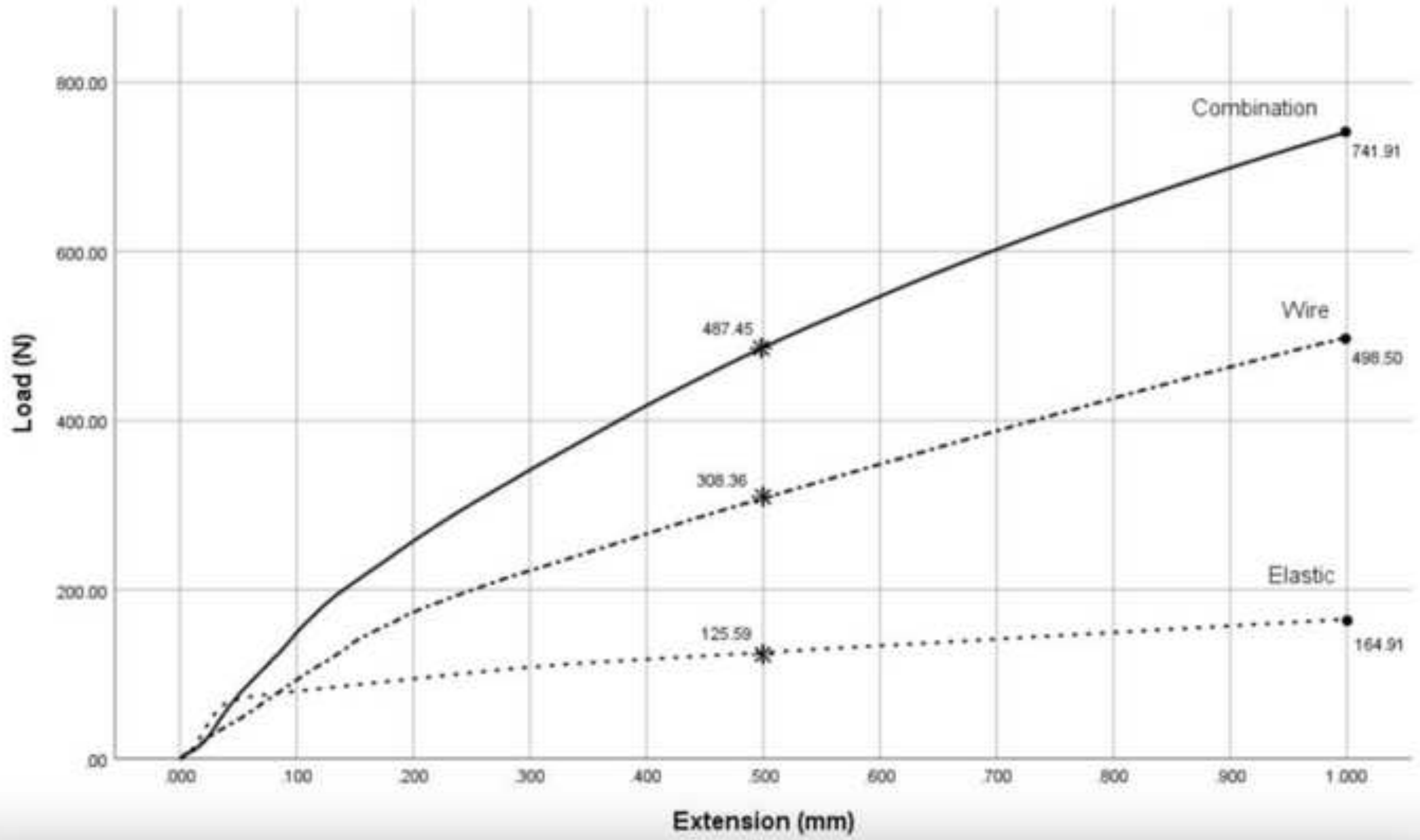

Click here to access/download
Supporting Information
Sudeep Pawa CV.pdf

Click here to access/download
Supporting Information
Supplemental File.pdf

Experimental Study

An In Vitro Study in Separating Tensile Loads during Maxillo-Mandibular Fixation using Wire and/or Elastics

Sudeep Pawa [1]¶, Surakit Visuttiwattanakorn [1]¶*

[1] Department of Oral and Maxillofacial Surgery, Faculty of Dentistry, Mahidol University, Bangkok, Thailand

* Corresponding author

Email: surakit_vis@mahidol.ac.th

Abstract. Intermaxillary fixation (IMF) or maxillo-mandibular fixation (MMF) is a fundamental process in stabilizing the maxilla and mandible through dental maximum intercuspation (MIP) during the management of trauma, orthognathic surgery, and reconstruction. Despite the availability of several techniques in achieving adequate maxillo-mandibular fixation, concerns have been raised regarding the sufficiency of using only latex elastics to counter displacing forces during reduction and fixation. ~~Intermaxillary fixation (IMF) or maxillo-mandibular fixation (MMF) is a technique that aims for the stabilization of the maxilla and mandible through the achievement of dental maximum intercuspation (MIP). It is considered a fundamental process in the management of facial trauma, reconstruction, and orthognathic surgery. Although there are various techniques for achieving adequate MMF, questions have risen about whether the use of only latex elastics for MMF is sufficient in opposing displacing forces during reduction and fixation.~~ An in vitro study was performed to determine which maxillo-mandibular fixation method between wire, elastics or a combination represents the greatest form of fixation using custom-made models stimulating dental arches. A loading rate of 1 mm/minute was used using an Instron 5566 Universal Testing Machine to separate the models unidirectionally in a vertical plane up to 1 mm apart. The tensile load was recorded at ~~both~~ 0.5 and 1 mm vertical separation. In each sample group, each process was repeated 10 times, and an average peak tensile load was recorded. A one-way ANOVA statistical analysis at $p<0.05$ significance level was conducted

Formatted: Font: (Default) Arial, 9 pt
Formatted: Normal
Formatted: Left: 1", Right: 1", Top: 1", Bottom: 1", Numbering: Continuous
Formatted: Font: (Default) Arial, 13 pt, Bold
Formatted: Font: (Default) Arial, 13 pt
Formatted: Font: (Default) Arial, 9 pt, Bold
Formatted: Font: (Default) Arial, 7.5 pt, Bold, Superscript
Formatted: Font: (Default) Arial, 7.5 pt, Superscript
Formatted: Font: (Default) Arial, 8 pt
Formatted: Font: (Default) Arial
Formatted: Font: (Default) Arial, 8 pt
Formatted: Font: (Default) Arial
Formatted: Font: (Default) Arial, 9 pt
Formatted: Normal, Line spacing: Double
Formatted: Normal, Indent: First line: 0.35", Line spacing: Double
Formatted: Font: 9 pt
Formatted: Font: (Default) Arial, 9 pt
Formatted: Font: 9 pt
Formatted: Font: (Default) Arial, 9 pt
Formatted: Font: 9 pt
Formatted: Font: (Default) Arial, 9 pt

and an outcome assessment showed statistical significance between all three subject groups. The average peak tensile load the combination group provided was greater than the wire and elastic groups for maximum maxillo-mandibular fixation.In this conducted in vitro study, a comprehensive analysis was performed to assess the ability of three distinct maxillo-mandibular fixation techniques to withstand opposing forces. The findings substantiate the superiority of the combination technique in achieving robust maxillo-mandibular fixation, positioning it as the most effective among the evaluated techniques.

Keywords: maxillo-mandibular fixation, intermaxillary fixation, tensile load

1. Introduction

Intermaxillary fixation (IMF) or maxillo-mandibular fixation (MMF) is a crucial technique that aims for the stabilization of the maxilla and mandible throughthrough the achievement of dental maximum intercuspation [1], particularly in cases of facial trauma, reconstruction, and orthognathic surgery [1]. . It is considered a fundamental process in the management of facial trauma, reconstruction, and orthognathic surgery. For example, during facial fractures involving the maxilla or mandible, maxillo-mandibular fixation (MMF) is used to adequately reduce fractured segments prior to internal fixation with plates and screws. Similarly, dDuring orthognathic surgery, surgeons apply maxillo-mandibular fixation at the intermediate stage of bimaxillary surgery or before applying rigid internal fixation [2,3]. Historically, traditional methods for achieving maxillo-mandibular fixation in dentate patients encompassed techniques like Erich's arch bars or interdental wiring.In the past, traditional techniques in dentate patients included devices such as arch bars and dental or interdental wiring with the most recent advancement being the introduction of intermaxillary fixation screws in 1989 [3,4]. The most recent introduction of intermaxillary fixation

Formatted: Font: 9 pt

Formatted: Font: (Default) Arial, Bold

Formatted: Font: (Default) Arial, 9 pt

Formatted: Line spacing: Double

Formatted: Font: (Default) Arial

Formatted: Font: (Default) Arial, 9 pt

Formatted: Font: (Default) Arial, 12 pt

Formatted: List Paragraph, Line spacing: Double, Numbered + Level: 1 + Numbering Style: 1, 2, 3, … + Start at: 1 + Alignment: Left + Aligned at: 0.25" + Indent at: 0.5"

Formatted: Indent: First line: 0"

Formatted: Font: (Default) Arial, 9 pt

Formatted: Font: 9 pt

Formatted: Font: (Default) Arial, 9 pt

Formatted: Font: 9 pt

Formatted: Font: (Default) Arial, 9 pt

Formatted: Font: 9 pt

Formatted: Font: (Default) Arial, 9 pt

Formatted: Font: 9 pt

Formatted: Font: (Default) Arial, 9 pt

Formatted: Font: 9 pt

Formatted: Font: (Default) Arial, 9 pt

Formatted: Font: 9 pt

Formatted: Font: (Default) Arial, 9 pt

Formatted: Font: 9 pt

Formatted: Font: (Default) Arial, 9 pt

Formatted: Font: 9 pt

Formatted: Right: 0.25"

Formatted: Position: Horizontal: Right, Relative to: Margin, Vertical: 0", Relative to: Paragraph, Wrap Around

screws in 1989 represented a notable advancement in this field [3,4]. The two most commonly used devices in orthognathic surgery include Mmaxillomandibular screws and orthodontic brackets with surgical hooks have become the two most commonly used techniques in orthognathic surgery [1,5,6]. In the past, maxillo-mandibular fixation was performed using wire ligatures, usually achieved with 26 or 24-gauge pre-stretched stainless steel stainless-steel wires which often caused discomfort, pain, or soft tissue damage for the patients during the immobilization period [1]. , [1]. However, due to the concerns about the potential source of needlestick injuries in the contaminated environment of the oral cavity which represents a health risk for surgeons and assistants, it is no longer considered the gold standard [2]. –

TodayIn response, orthodontic latex elastics are used as an alternative to wire ligatures [7]. A recent study showed that heavy orthodontic elastics had a force decay of 31% after 24 hours in simulated oral environments [6]. Statistical analysis showed statistical significance in force reduction in the first 24 hours however, force decay between 1-14 days was not significant [6]. Evidence suggests elastics offer adequate traction even in scenarios where the use of wires may be limited [2,6,7,8]. The goal of mMaxillo-mandibular fixation should be ableis to resist displacing forces whilst the new position of the alveolus or bony segments is established and fixed with rigid internal fixation [2,9]. Despite the importance of these techniques, a significant research gap exists. Inadequate intermaxillary fixation (IMF) following oral and maxillofacial procedures can lead to malocclusion, delayed healing, bone nonunion or malunion, infections, nerve damage, aesthetic issues, functional difficulties, and chronic pain. Proper alignment and immobilization are crucial to prevent these complications and ensure successful recovery and outcomes. Although, multiple methods for achieving optimal maxillo-mandibular fixation have been proposed and used, no prior lab or clinical investigation has comprehensively examined the tensile loads applied during the application of separating forces in various maxillo-mandibular fixation techniques. The primary objective of this

Formatted: Font: (Default) Arial, 9 pt
Formatted: Font: 9 pt
Formatted: Font: (Default) Arial, 9 pt
Formatted: Font: 9 pt
Formatted: Font: (Default) Arial, 9 pt
Formatted: Font: (Default) Arial, 9 pt, Not Bold
Formatted: Font: (Default) Arial, 9 pt
Formatted: Font: 9 pt
Formatted: Font: 9 pt
Formatted: Font: (Default) Arial, 9 pt
Formatted: Font: 9 pt
Formatted: Font: (Default) Arial, 9 pt
Formatted: Font: 9 pt
Formatted: Font: (Default) Arial, 9 pt
Formatted: Font: 9 pt
Formatted: Font: (Default) Arial, 9 pt
Formatted: Font: 9 pt
Formatted ...
Formatted ...
Formatted ...
Formatted ...
Formatted ...
Formatted ...
Formatted ...
Formatted ...
Formatted ...
Formatted ...
Formatted ...
Formatted ...
Formatted ...
Formatted ...
Formatted ...
Formatted ...
Formatted ...
Formatted ...

study is to determine which maxillo-mandibular fixation method between wire, elastics, and combination represents the most effective form of maxillo-mandibular fixation method. By providing numerical evidence of distinct tensile loads associated with different maxillo-mandibular fixation techniques, this study aims to address this critical knowledge gap and contribute to the advancement of clinical practices in this domain.~~Although there are various techniques for achieving adequate maxillo-mandibular fixation, questions have risen about whether the use of latex elastics for maxillo-mandibular fixation is sufficient in opposing displacing forces during reduction and fixation.~~

2. Materials and Methods

Two models as ~~shown~~ depicted in Fig~~ure~~ ~~–~~1 ~~were~~ were designed and fabricated using Siemens NX CAD using computer-aided design and manufacturing with stainless steel ~~to imitate a maxillary and mandibular arch~~. The models were meticulously designed and fabricated to closely replicate both the maxillary and mandibular arches, consisting of seven screws representing seven teeth in each quadrant. Custom-milled screws as ~~shown~~ illustrated in Fig~~ure~~ 2 were fabricated using a wire cut technique to mimic maxillo-mandibular screws for the fixation of ligature wire (Stainless steel 0.020” Gauge 24, Lot No. 14662939, Fort Wayne Metals, Indiana, USA) and ortho-dontic latex elastics (Ram Zoo Pak Elastics, 6 Oz/170g, ¼”, 6.35 mm, Lot No. 082091864, Ormco Corporation, California, USA). This experimental study allowed for a detailed investigation into the mechanics and outcomes of various fixation methods, shedding light on the performance characteristics of wire ligatures and orthodontic latex elastics in the context of maxillo-mandibular fixation.

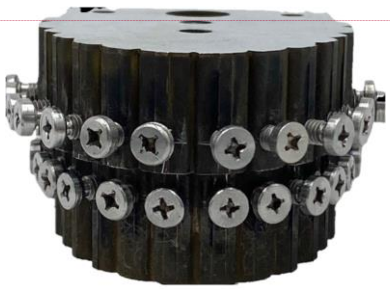

Formatted: Font: 9 pt

Formatted: Font: (Default) Arial, 9 pt

Formatted: Font: (Default) Arial, 9 pt, Bold

Formatted: Font: (Default) Arial, 12 pt

Formatted: List Paragraph, Line spacing: Double, Numbered + Level: 1 + Numbering Style: 1, 2, 3, … + Start at: 1 + Alignment: Left + Aligned at: 0.25" + Indent at: 0.5"

Formatted: Font: 9 pt

Formatted: Font: 9 pt

Formatted: Indent: First line: 0"

Formatted: Font: 9 pt

Formatted: Font: 9 pt

Formatted: Right: 0.25"

Formatted: Position: Horizontal: Right, Relative to: Margin, Vertical: 0", Relative to: Paragraph, Wrap Around

Fig 1. Custom-made dental models through CAD/CAM technique

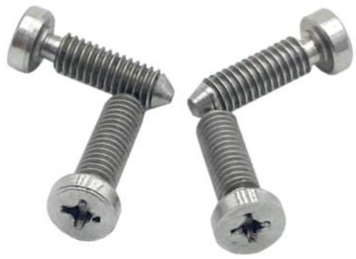

Fig 2. Screws designed and fabricated to represent IMF screws and orthodontic brackets

Formatted: Font: 9 pt, Bold

Formatted: Font: 9 pt

Formatted: Normal, Line spacing: Double

Formatted: Normal, Line spacing: Double

Formatted: Right: 0.25"

Formatted: Position: Horizontal: Right, Relative to: Margin, Vertical: 0", Relative to: Paragraph, Wrap Around

Figure 1. Picture of the customized dental models

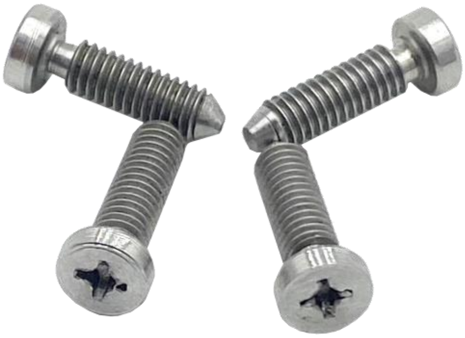

Figure 2. Screws were designed and fabricated using a wire cut technique to represent IMF screws and orthodontic brackets.

A pilot study was conducted to determine the most stable fixation technique between different fixation patterns in both the wire and elastic groups as shown in Appendix A.In order to ascertain the optimal fixation technique, a preliminary pilot study was carried out. This preliminary investigation aimed to identify the most stable fixation pattern among various fixation patterns, as depicted in Appendix A (Supplemental File). From the pilot studyUpon analyzing the outcomes of the pilot study, the interlocking with an overlapping elastic group (E1) and the overlapping box wire group (W2) showed the highest ability to resist displacing forces when tensile loads waswere applied and was therefore chosen as sample groups were selected as the representative sample groups representing wire and elastic techniques in this study.

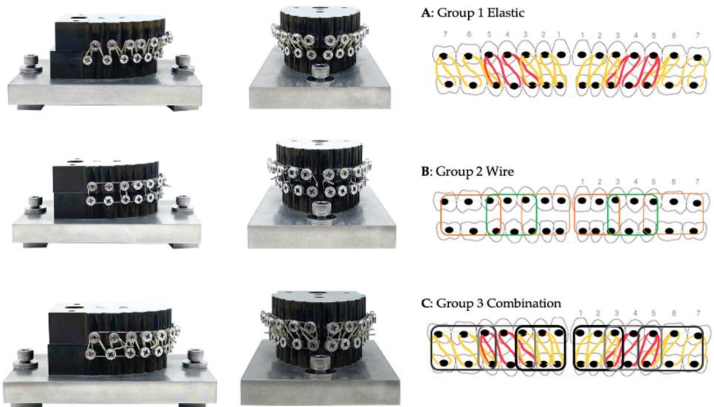

Formatted: Normal, Indent: Left: 0", Line spacing: Double

Formatted: Normal, Line spacing: Double

Formatted: Normal, Indent: First line: 0.25", Line spacing: Double

Formatted: Font: 9 pt

Formatted: Font: 9 pt

Formatted: Font: 9 pt

Formatted: Right: 0.25"

Formatted: Position: Horizontal: Right, Relative to: Margin, Vertical: 0", Relative to: Paragraph, Wrap Around

Formatted: Font: 9 pt

Formatted: Indent: First line: 0"

Formatted: Indent: First line: 0"

Fig 3 (A, B, C). Different MMF techniques representing all 3 sample groups

Formatted: Font: 9 pt, Not Bold, (Asian) Chinese (PRC)

Formatted: Font: 9 pt

Formatted: Normal, Indent: Left: 0.35", First line: 0.35", Line spacing: Double

Formatted: Normal, Line spacing: Double

Formatted: Font: (Default) Arial, 9 pt

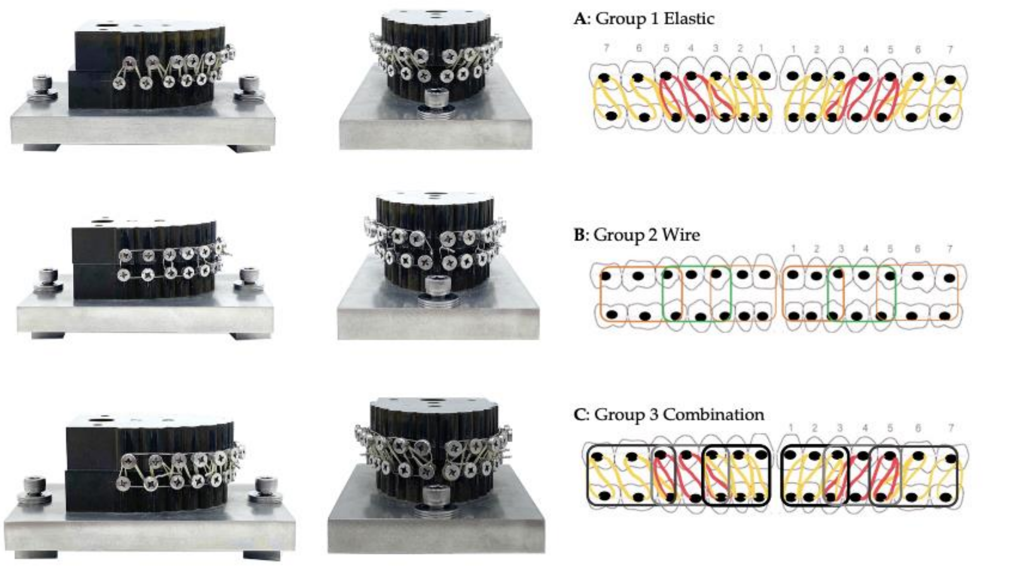

Right: 0.25"

Position: Horizontal: Right, Relative to: Margin, Vertical: 0", Relative to: Paragraph, Wrap Around

Fig (A, B, C). Different MMF techniques representing all 3 sample groups.

The study’s design comprised of three3 sample groups which consistedconsisting of wire (Figure 3B), elastic (Figure 3A), and the a combination group (Figure 3C); which combined the fixation pattern of both the wire and elastic groups. Sample size calculation using Stata/SE 15.1 for power analysis for one-way analysis of variance, with statistical significance set at 0.001 and a power of 0.99, was calculated using data from the pilot study. According to the calculation, n=4 pera minimum sample group of 4, group was established. However, for this particular study, a larger sample group of 10 was employed. In this study, n=10 was used. The samples were tested using a 1000 Newton load cell using the Instron 5566 Universal Testing Machine (Instron Ltd., Buckinghamshire, England) (DT-40-73) Universal Testing Machine, Maxillomandibular fixation was achieved executed in each sample according to the sample groupspecified sample group parameters and loaded on and tested separated using the universal testing machine until 0.5 mm and 1 mm of vertical separation between the upper and lower models was achieved

Formatted: Normal, Indent: Left: 0", First line: 0", Line spacing: Double
Formatted: Normal, Line spacing: Double
Formatted: Font: 9 pt
Formatted: Font: (Default) Arial, 9 pt, (Asian) Chinese (PRC)
Formatted: Font: 9 pt
Formatted: Font: (Default) Arial, 9 pt, (Asian) Chinese (PRC)
Formatted: Font: 9 pt
Formatted: Font: (Default) Arial, 9 pt, (Asian) Chinese (PRC)
Formatted: Font: 9 pt
Formatted: Font: (Default) Arial, 9 pt, (Asian) Chinese (PRC)
Formatted: Font: 9 pt
Formatted: Font: (Default) Arial, 9 pt, (Asian) Chinese (PRC)
Formatted: Font: 9 pt
Formatted: Font: (Default) Arial, 9 pt, (Asian) Chinese (PRC)
Formatted: Font: 9 pt
Formatted: Font: (Default) Arial, 9 pt, (Asian) Chinese (PRC)
Formatted: Font: 9 pt
Formatted: Font: (Default) Arial, 9 pt, (Asian) Chinese (PRC)
Formatted: Font: 9 pt
Formatted: Font: (Default) Arial, 9 pt, (Asian) Chinese (PRC)
Formatted: Font: 9 pt
Formatted ...
Formatted: Font: 9 pt
Formatted ...
Formatted: Right: 0.25"
Formatted ...

as ~~shown~~ demonstrated in Fig~~ure~~ 4A, 4B, 4C. Each process was repeated 10 times and the average peak tensile load was recorded and plotted.

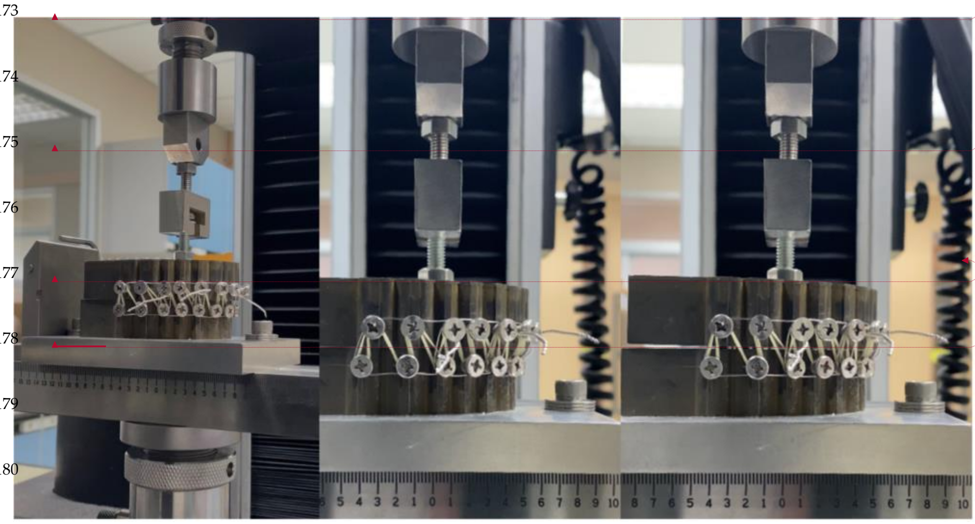

Fig 4A **Fig 4B** **Fig 4C**

Fig 4A. Orientation of dental models on the Instron 5566 Universal Testing Machine.
Fig 4B. Models at rest position at 0 mm separation.
Fig 3C. Models at complete 1 mm separation (complete cycle)

Formatted: Font: 9 pt
Formatted: Font: (Default) Arial, 9 pt, (Asian) Chinese (PRC)
Formatted: Font: 9 pt
Formatted: Font: 9 pt
Formatted: Font: (Default) Arial, 9 pt
Formatted: Normal, Line spacing: Double
Formatted: Font: 9 pt
Formatted: Font: 9 pt, Bold
Formatted: Font: 9 pt
Formatted: Left, Indent: Left: 0.71", First line: 0.35", Line spacing: single
Formatted: Font: 9 pt, Not Bold
Formatted: Font: 9 pt
Formatted: Font: 9 pt, Not Bold
Formatted: Font: 9 pt
Formatted: Font: 9 pt, Not Bold, (Asian) Chinese (PRC)
Formatted: Font: (Default) Arial, 9 pt
Formatted: Normal, Indent: Left: 0.71", First line: 0.35"
Formatted: Normal, Line spacing: Double
Formatted: Font: (Default) Arial, 9 pt
Formatted: Right: 0.25"
Formatted: Position: Horizontal: Right, Relative to: Margin, Vertical: 0", Relative to: Paragraph, Wrap Around

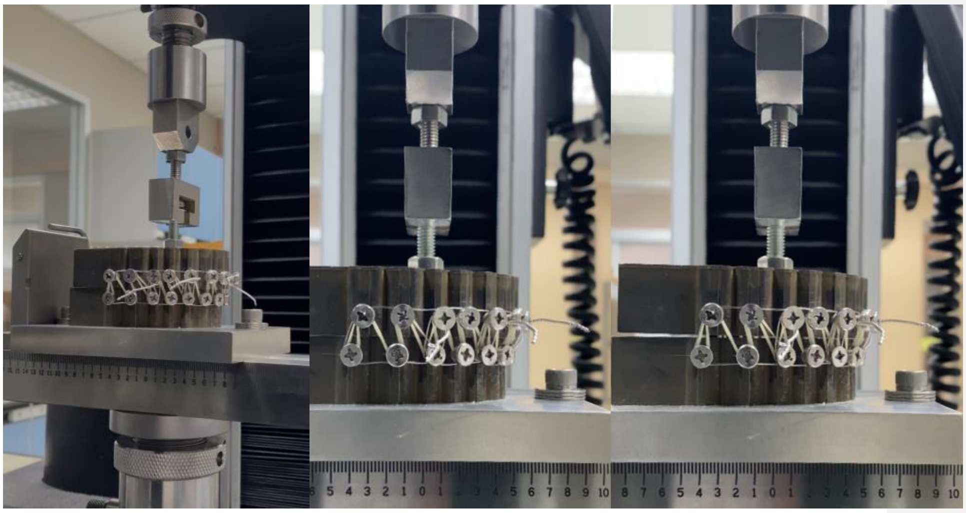

Formatted: Normal, Indent: Left: 0", First line: 0", Line spacing: Double

Formatted: Font: (Default) Arial, 9 pt

Formatted: Right: 0.25"

Formatted: Position: Horizontal: Right, Relative to: Margin, Vertical: 0", Relative to: Paragraph, Wrap Around

~~**Figure 4A** **Figure 4B** **Figure 4C**~~

~~**Figure 4A.** Orientation of dental models on the Instron 5566 Universal Testing Machine~~

~~**Figure 4B.** Models at rest position at 0 mm separation~~

~~**Figure 4C**. Models at complete 1 mm separation (Complete Cycle)~~

Descriptive statistics ~~were~~ was calculated using Microsoft Excel and SPSS (Statistical Package for the Social Sciences) version 25 (IBM, Chicago, IL, USA). Data ~~were~~ was analyzed using a one-way ANOVA and ~~statistical differences~~multiple comparisons between each group ~~were~~ was evaluated for statistical significance using Bonferroni's Test. The level of significance was set at $p<0.05$. A comparison between all 3 groups is shown in Table 1 ~~Table 1~~.

Formatted: Font: (Default) Arial, 9 pt

Formatted: Normal, Line spacing: Double

Formatted: Normal, Indent: Left: 0", First line: 0", Line spacing: Double

Formatted: Font: 9 pt

Formatted: Font: (Default) Arial, 9 pt

Formatted: Font: 9 pt

Formatted: Font: (Default) Arial, 9 pt

Formatted: Font: 9 pt

Formatted: Font: (Default) Arial, 9 pt

Formatted: Font: 9 pt

Formatted: Font: (Default) Arial, 9 pt

Formatted: Font: 9 pt

Formatted: Font: (Default) Arial, 9 pt

Formatted: Font: 9 pt

Formatted: Font: (Default) Arial, 9 pt

Formatted: Font: 9 pt

Formatted: Line spacing: Double

Formatted: Font: 9 pt

Formatted: Right: 0.25"

Formatted: Position: Horizontal: Right, Relative to: Margin, Vertical: 0", Relative to: Paragraph, Wrap Around

Table 1 Comparing tensile loads using elastic, wire, and combination methods

	Elastic	Wire	Combination	P-value
Load (N): 1 mm	164.91±11.25	498.50±24.37	741.91±34.90	<0.001*
Load (N): 0.5 mm	125.59±8.22	308.36±18.67	487.45±31.98	<0.001*

Data were expressed as mean±SD and analyzed with One-way ANOVA

*Statistically significant at the 0.05 level

Table 1 Comparing Tensile Load Using Elastic, Wire and Combination Methods

	Elastic	Wire	Combination	P-value
Load (N): 1 mm	164.91±11.25	498.50±24.37	741.91±34.90	<0.001*
Load (N): 0.5 mm	125.59±8.22	308.36±18.67	487.45±31.98	<0.001*

Data were expressed as mean±SD and analyzed with One-way ANOVA

*Statistically significant at the 0.05 level

3. ~~3.~~ Results

This study showed that the average tensile load to achieve 1 mm of vertical separation of the models in the elastic group was at 164.91±11.25 N, the wire group at 498.50±24.37 N, and the combination group at 741.91±34.90 N (Fig 5~~ure 5~~). Statistical analyses using one-way ANOVA showed the average tensile load difference between all 3 groups was statistically significant at the 0.05 level (p<0.001) (~~Table 1~~Table 1, Fig 5~~, Figure 5~~).

Multiple comparisons ~~were~~ was therefore performed using Bonferroni's test to evaluate the statistical difference among the 3 groups. The mean difference in tensile load to resist 1 mm separation between the wire and elastic groups was statistically significant at the 0.05 level (p<.001). A mean difference in tensile load was 333.59 N (95%CI

Formatted: Font: 9 pt

Formatted: Font: 9 pt

Formatted: Font: 9 pt

Formatted: Font: 9 pt

Formatted: Line spacing: single

Formatted: Font: (Default) Arial, 9 pt

Formatted: Normal

Formatted: Normal, Indent: Left: 0"

Formatted: Font: (Default) Arial, 9 pt, Bold

Formatted: Font: (Default) Arial, 12 pt

Formatted: List Paragraph, Line spacing: Double, Numbered + Level: 1 + Numbering Style: 1, 2, 3, … + Start at: 1 + Alignment: Left + Aligned at: 0.25" + Indent at: 0.5"

Formatted: Indent: First line: 0"

Formatted: Font: (Default) Arial, 9 pt

Formatted: Font: 9 pt

Formatted: Font: (Default) Arial, 9 pt

Formatted: Font: 9 pt

Formatted: Font: (Default) Arial, 9 pt

Formatted: Font: 9 pt

Formatted: Font: (Default) Arial, 9 pt

Formatted: Font: 9 pt

Formatted: Normal, Indent: First line: 0.25", Line spacing: Double

Formatted: Font: (Default) Arial, 9 pt

Formatted: Font: 9 pt

Formatted: Font: (Default) Arial, 9 pt

Formatted: Right: 0.25"

Formatted: Position: Horizontal: Right, Relative to: Margin, Vertical: 0", Relative to: Paragraph, Wrap Around

304.57, 362.61), exhibiting that the wire group had an average tensile load 3.02 times greater than that of the elastic group. When comparing the combination group to the elastic group, the statistical analysis also showed significance at the 0.05 level (p<.001). A mean difference ~~in~~ in tensile load was at 576.99 N (95%CI 547.98, 606.02) or 4.50 times greater than the elastic group (Fig~~ure 5~~5). Lastly, when comparing the combination group to the wire group, statistical significance was also reported showing a mean difference in tensile load at 243.91 N (95%CI 214.39, 272.43), showing the combination group had an average tensile load to be 1.49 times greater than the average load of the wire group (~~Table 2, Figure 5~~Table 2, Fig 5).

Table 2 Multiple comparison between sample groups

**Group vs. Group**	**Mean difference (95%CI)**	**Standard error**	**P-value**
**Load (N): Extension 1 mm**			
Wire vs. Elastic	333.59 (304.57, 362.61)	11.37	<0.001*
Combination vs. Elastic	576.99 (547.98, 606.02)	11.37	<0.001*
Combination vs. Wire	243.41 (214.39, 272.43)	11.37	<0.001*
**Load: Extension 0.5 mm (N)**			
Wire vs. Elastic	182.77 (157.77, 207.76)	9.79	<0.001*
Combination vs. Elastic	361.85 (336.86, 386.85)	9.79	<0.001*
Combination vs. Wire	179.09 (154.09, 204.08)	9.79	<0.001*

Multiple comparisons were analyzed with Bonferroni method

* The mean difference is significant at the 0.05 level

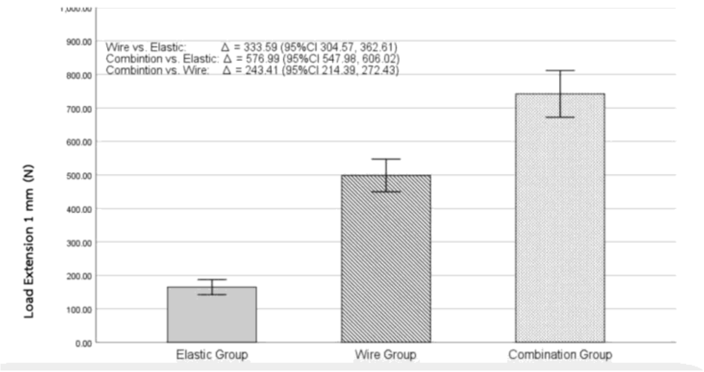

Formatted: Font: 9 pt
Formatted: Font: (Default) Arial, 9 pt
Formatted: Font: 9 pt
Formatted: Font: (Default) Arial, 9 pt
Formatted: Font: 9 pt
Formatted: Font: (Default) Arial, 9 pt
Formatted: Font: 9 pt
Formatted: Font: 9 pt
Formatted: Indent: First line: 0"
Formatted Table
Formatted: Font: 9 pt
Formatted: Font: 9 pt
Formatted: Font: 9 pt
Formatted: Font: 9 pt
Formatted: Font: 9 pt
Formatted: Font: 9 pt
Formatted: Font: 9 pt
Formatted: Font: 9 pt
Formatted: Font: 9 pt
Formatted: Font: 9 pt
Formatted: Font: 9 pt
Formatted: Font: 9 pt
Formatted: Right: 0.25"
Formatted: Position: Horizontal: Right, Relative to: Margin, Vertical: 0", Relative to: Paragraph, Wrap Around

Table 2 Multiple comparison between sample groups

Group vs. Group	Mean difference (95%CI)	Standard error	P-value
**Load (N): Extension 1 mm**			
Wire vs. Elastic	333.59 (304.57, 362.61)	11.37	<0.001*
Combination vs. Elastic	576.99 (547.98, 606.02)	11.37	<0.001*
Combination vs. Wire	243.41 (214.39, 272.43)	11.37	<0.001*
**Load (N): Extension 0.5 mm**			
Wire vs. Elastic	182.77 (157.77, 207.76)	9.79	<0.001*
Combination vs. Elastic	361.85 (336.86, 386.85)	9.79	<0.001*
Combination vs. Wire	179.09 (154.09, 204.08)	9.79	<0.001*

Multiple comparisons were analyzed with Bonferroni method

*The mean difference is significant at the 0.05 level

Fig 5. Mean tensile loads of maxillo-mandibular fixation between all 3 groups at 1 mm separation

Formatted: Font: (Default) Arial, 9 pt

Formatted: Normal, Line spacing: Double

Formatted: Normal, Indent: First line: 0.25", Line spacing: Double

Formatted: Font: 9 pt

Formatted: Font: 9 pt, Not Bold, (Asian) Chinese (PRC)

Formatted: Font: 9 pt, (Asian) Chinese (PRC)

Formatted: Font: 9 pt, Not Bold, (Asian) Chinese (PRC)

Formatted: Font: 9 pt, (Asian) Chinese (PRC)

Formatted: Font: (Default) Arial, 9 pt

Formatted: Normal, Line spacing: Double

Formatted: Font: 9 pt

Formatted: Font: (Default) Arial, 9 pt

Formatted: Right: 0.25"

Formatted: Position: Horizontal: Right, Relative to: Margin, Vertical: 0", Relative to: Paragraph, Wrap Around

Formatted: Normal, Indent: Left: 0", Line spacing: Double

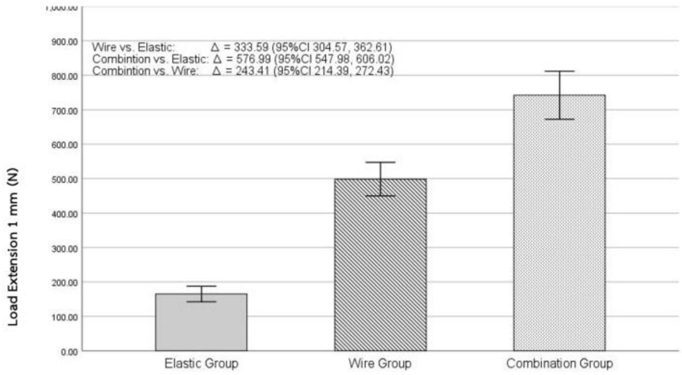

...tted: Right: 0.25"

...tted: Position: Horizontal: Right, Relative ...rgin, Vertical: 0", Relative to: Paragraph, Around

Figure 5. Mean tensile load of maxillo-mandibular fixation between all 3 groups at 1 mm separatio n.

A Sstatistical analyseis of the same subject groups waswere also performed evaluating the separation of the dental models at 0.5 mm using the same study parameters. The average tensile load in the elastic group was 125.59±8.22 N, the wire group at 308.36±18.67 N, and the combination group at 487.45±31.98 N, respectively (Figure 66). Statistical analysis using one-way ANOVA showed the average tensile load difference between all 3 groups was statistically significant at 0.05 (p<0.001) (Table 1Table 1). Multiple comparisons were therefore performed using Bonferroni's test to evaluate the statistical difference between all 3 groups. The mean difference in tensile load to resist 0.5 mm separation between the wire group compared to the elastic was statistically significant at the 0.05 level (p<0.001). A mean difference in tensile load was 182.77 N (95%CI 157.77, 207.76), displaying that the wire group had an average tensile load 2.46 times greater than that of the elastic group. When comparing the combination group to the elastic group, the statistical analysis also showed significance at the 0.05 level (p<0.001). A mean difference in tensile load was at 361.85 N (95%CI 336.86, 386.85) or the combination group could resist displacing forces 3.88 times that of the elastic group. Lastly, when comparing the combination group to the wire group, statistical significance was also reported showing a mean difference in tensile load at 179.09 N (95%CI 154.09, 204.08), meaning the combination group had an average tensile load to be 1.58 times the average load of the wire group (Table 2, Figure 6Table 2, Fig 6). The data from this study was therefore plotted as shown in Fig 7.

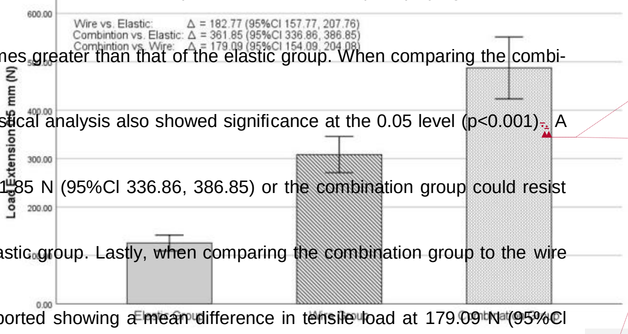

Formatted: Font: (Default) Arial, 9 pt
Formatted: Normal, Line spacing: Double
Formatted: Font: (Default) Arial, 9 pt
Formatted: Font: (Default) Arial, 9 pt
Formatted: Normal, Indent: Left: 0", First line: 0", Line spacing: Double
Formatted: Font: 9 pt
Formatted: Font: 9 pt
Formatted: Font: (Default) Arial, 9 pt
Formatted: Font: 9 pt
Formatted: Font: (Default) Arial, 9 pt
Formatted: Font: 9 pt
Formatted: Font: (Default) Arial, 9 pt
Formatted: Font: 9 pt
Formatted: Font: (Default) Arial, 9 pt
Formatted: Font: 9 pt
Formatted: Font: (Default) Arial, 9 pt
Formatted: Font: 9 pt
Formatted: Right: 0.25"
Formatted: Position: Horizontal: Right, Relative to: Margin, Vertical: 0", Relative to: Paragraph, Wrap Around

showing a line graph that demonstrates the differences in tensile loads between all 3 sample groups at 0.5 and 1 mm vertical separation.

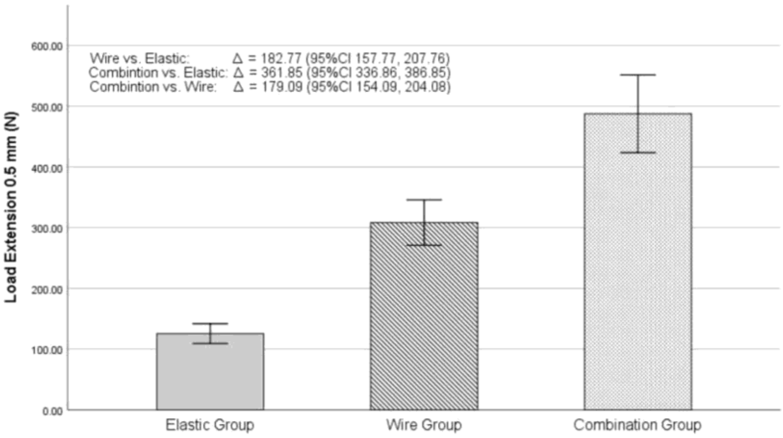

Fig 6. Mean tensile loads of maxillo-mandibular fixation between at 3 groups at 0.5 mm separation

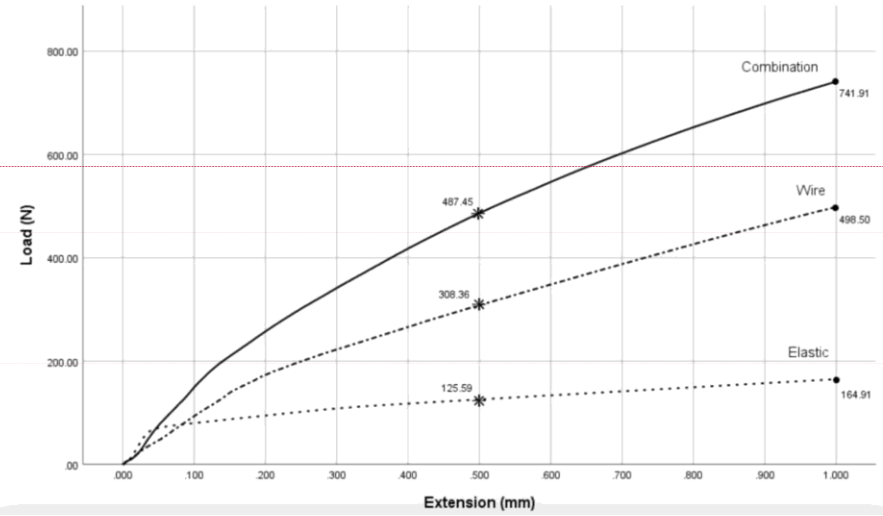

Formatted: Font: (Default) Arial, 9 pt
Formatted: Font: 9 pt
Formatted: Font: (Default) Arial, 9 pt
Formatted: Normal, Indent: Left: 0", Line spacing: Double
Formatted: Font: 9 pt
Formatted: Normal, Indent: Left: 0", First line: 0", Line spacing: Double
Formatted: Font: (Default) Arial, 9 pt
Formatted: Font: 9 pt
Formatted: Font: (Default) Arial, 9 pt
Formatted: Normal, Indent: Left: 0", First line: 0", Line spacing: Double
Formatted: Font: 9 pt
Formatted: Right: 0.25"
Formatted: Position: Horizontal: Right, Relative to: Margin, Vertical: 0", Relative to: Paragraph, Wrap Around

Fig 7. Line graph showing the average tensile loads at both 0.5 and 1 mm separation

Figure 6. Bar chart presenting the mean tensile load between maxillo-mandibular fixation using elastic, wire, and combination groups at 0.5 mm separation.

4. 4. Discussion and Conclusion

In this conducted in vitro study, a comprehensive analysis was performed to assess the ability of three distinct maxillo-mandibular fixation techniques to withstand opposing forces. These techniques comprised of the elastic group, the wire group, and the combination group. Statistical significance was observed in this comparison, yielding valuable insights into their relative efficacy. In this in vitro study, there was statistical significance when comparing the ability to resist opposing forces when using 3 different maxillo-mandibular fixation techniques, comprising of an elastic group, wire group, and combination group. The authors found that the combination group could resist forces up to 741 N, the wire group up to 498 N, and the elastic group up to 164 N before causing a 1 mm separation between the dental models. Data shows that the best fixation technique was the combination group followed by the wire group and lastly the elastic group, respectively. When comparing the average tensile loads that all 3 groups could resist before causing a separation between the dental models of at least 0.5 mm, the authors found that the elastic group was the least resistant to opposing forc esWhen analyzing the average tensile loads that each of these groups could endure before leading to a separation of at least 0.5 mm between the dental models, the elastic group emerged as the least resistant to opposing forces.. The data further highlights a substantial discrepancy between the group with highest force-resisting capability (the combination group) and the group with the lowest

Formatted: Font: 9 pt, Bold, (Asian) Chinese (PRC)

Formatted: Font: 9 pt, (Asian) Chinese (PRC)

Formatted: Font: (Default) Arial, 9 pt

Formatted: Normal, Indent: Left: 0", First line: 0", Line spacing: Double

Formatted: Font: (Default) Arial, 12 pt

Formatted: Font: 12 pt

Formatted: Font: 16 pt

Formatted: Font: (Default) Arial, 12 pt

Formatted: Font: (Default) Arial, 16 pt

Formatted: Font: 9 pt

Formatted: Font: (Default) Arial, 9 pt

Formatted: Font: 9 pt

Formatted: Font: (Default) Arial, 9 pt

Formatted: Font: 9 pt

Formatted: Font: (Default) Arial, 9 pt

Formatted: Font: 9 pt

Formatted: Right: 0.25"

Formatted: Position: Horizontal: Right, Relative to: Margin, Vertical: 0", Relative to: Paragraph, Wrap Around

(the elastic group). This disparity was significant, revealing a striking 400% difference in their respective abilities to withstand opposing forces. These findings substantiate the superiority of the combination technique in achieving robust maxillo-mandibular fixation, positioning it as the most effective among the evaluated techniques. Using data from both sample studies, it was found that between the group that was most capable of resisting forces (Combination Group) and the group least capable of resisting forces (Elastic Group), there was at least a 400% difference in the capability of resisting forces. From this study, it can be concluded that the combination technique could resist forces at least 1.5x or 150% more when compared to the wire group and at least 4x or 400% more when compared to the elastic group as shown in Figure 7.

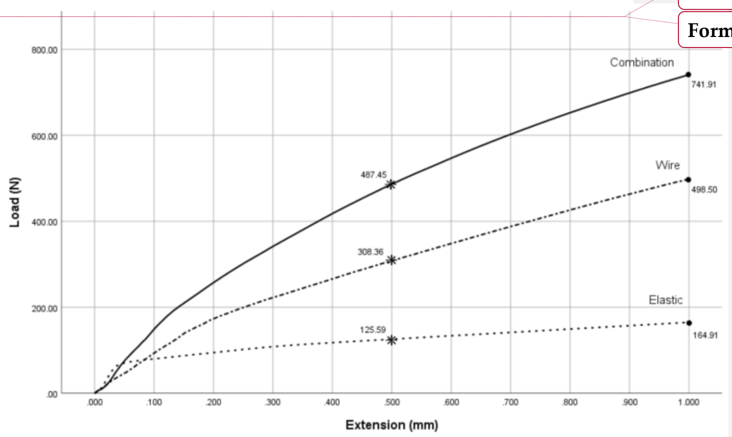

Figure 7. Line graph showing the average tensile loads at both 0.5 and 1 mm separation.

A.T. Smith in 1993 concluded that traditional wiring was a potential source of needlestick injuries in a contaminated environment and represented a health risk to surgeons and other healthcare providers. A systematic review by

Formatted: Font: (Default) Arial, 9 pt

Formatted: Font: (Default) Arial, 9 pt, Bold

Formatted: Font: (Default) Arial, 9 pt

Formatted: Font: (Default) Arial, 9 pt, Bold

Formatted: Font: (Default) Arial, 9 pt

Formatted: Font: (Default) Arial, 9 pt, Bold

Formatted: Font: (Default) Arial, 9 pt

Formatted: Right: 0.25"

Formatted: Position: Horizontal: Right, Relative to: Margin, Vertical: 0", Relative to: Paragraph, Wrap Around

Bouya et al. in 2020 found the global prevalence of needle stick injuries, which included incidences caused by wires intraoperatively, was as high as 2 million annually in healthcare workers. Even though the use of elastics could reduce the risk of needlestick injuries, in vitro data from this study can conclude that using only elastics in achieving maxilla-mandibular fixation was insufficient in resisting opposing forces when compared to the wire and combination groups.In 1993, A.T. Smith highlighted the potential risk of needlestick injuries associated with traditional wiring in contaminated environments, posing health hazards to healthcare providers. A systematic review by Bouya et al. in 2020 demonstrated the alarming global prevalence of needlestick injuries, some of which were attributed to intraoperative wire-related incidents, affecting as many as 2 million healthcare workers annually. While elastics could mitigate the needlestick risk, our in vitro study data establishes that relying solely on elastics for maxillo-mandibular fixation is insufficient in resisting opposing forces when compared to the wire and combination techniques.

A study in 1970 by Yildirim studied the maximum opening and closing forces exerted by different skeletal types. The sample groups consisted of skeletal open and closed bites. The study found that there was no statistical difference between opening forces between the two groups. The average opening forces measured in the study were equivalent to 137-169 N [11]. Whereas a study by Brunton et al. in 2018 with 149 patients aged 20-60 years with overall good and general health found that in a large sample of participants of a broad age range and a demographically diverse background, jaw opening forces were greater in males than females. Men had greater maximum opening forces with median values of —157.77 N compared to women at 166.61 N [12]. Our in vitro study data sheds light on maxillo-mandibular fixation using elastics alone. It demonstrates that elastics can only resist tensile loads causing a 0.5 mm separation of dental models at 126 N and a 1 mm separation at 165 N which is insufficient to resist the maximum opening forces in men and women as studied by Yilridum and Brunton et al.

Formatted: Font: 9 pt

Formatted: Font: 9 pt

Formatted: Font: (Default) Arial, 9 pt

Formatted: Font: (Default) Arial

Formatted: Font: (Default) Arial, 9 pt

Formatted: Font: 9 pt

Formatted: Font: 9 pt

Formatted: Font: 9 pt

Formatted: Right: 0.25"

Formatted: Position: Horizontal: Right, Relative to: Margin, Vertical: 0", Relative to: Paragraph, Wrap Around

Transitioning to clinical scenarios, we reviewed the maximum push force that can be exerted through human force, particularly relevant during the surgical phase when applying temporary maxillo-mandibular fixation application or fracture reduction phases. Das et al. in 2004 examined isometric pull and push strength profiles in both genders and across different positions. Notably, the study found that maximum push strength in males ranged from 140 N to 227 N, depending on the seated or standing position. In females, the values ranged from 96 N to 140 N, again based on seating and standing positions [13].

This comprehensive examination of prior research and our in vitro findings collectively underscores the intricate interplay between technique efficacy, anatomical factors, and the practical forces encountered in clinical scenarios, providing a nuanced perspective on maxillo-mandibular fixation and its potential clinical implications.

Data from this in vitro study shows that maxillo-mandibular fixation from elastic only can resist tensile loads causing a 0.5 mm separation of the dental models at 126 N and a 1 mm separation at 165 N. Other than maximum opening forces in human subjects, the authors also reviewed the maximum push force that can be exerted through human force. Intraoperatively, especially during the phase of applying temporary maxillomandibular fixation or during the reduction of fractured segments, surgeons apply some forces that may cause instability and separation of occlusal contacts even in absolute maxillomandibular fixation. Das et al. 2004 conducted a study measuring the isometric pull and push strength profiles in males and females in both a seated and standing positions. The study found that the maximum push strength in males was 227 N in the seated position and 140 N in the standing position. Whereas in females, the maximum push strength was 96 N in a seated position and 140 N in a standing position [13]. Therefore, a conclusion can be made after reviewing the maximum opening forces exerted by human force and maximum push force exerted in both a standing and sitting position that using elastic alone for maxillomandibular fixation cannot achieve adequate stability sufficient in opposing displacing forces during reduction and fixation. As

Formatted: Font: 9 pt

Formatted: Indent: First line: 0.25"

Formatted: Font: 9 pt

Formatted: Font: (Default) Arial, 9 pt

Formatted: Right: 0.25"

Formatted: Position: Horizontal: Right, Relative to: Margin, Vertical: 0", Relative to: Paragraph, Wrap Around

this study is a pilot study and the first of its kind to study the tensile load from maxillo-mandibular fixation, additional studies may be needed before a clinical guideline can be made. This study represents an in vitro study performed using an Instron 5566 Universal Testing Machine where the dental models were vertically displaced. In reality, opposing forces between the mandible and maxilla may not only be in the vertical and/or horizontal dimensions but may consist of a multi-directional force. Additional studies may need to be performed to better understand the behavior of the dental models when separation forces were applied multi-directionally.

5. Conclusion

From this study, it can be established that maxillomandibular fixation using the combination technique could resist forces at least 1.5x or 150% more when compared to the wire group and at least 4x or 400% more when compared to the elastic group as shown in Fig 7.

Furthermore, a conclusion can be made after reviewing the maximum opening forces exerted by human force and maximum push force exerted in both a standing and sitting position that using elastic alone for maxillomandibular fixation cannot achieve adequate stability sufficient in opposing displacing forces during reduction and fixation. As this study is a pilot study and the first of its kind to study the tensile load from maxillo-mandibular fixation, additional studies may be needed before a clinical recommendation can be made. This study, conducted in a controlled in vitro environment utilizing the Instron 5566 Universal Testing Machine to vertically displace dental models, provides an initial understanding of tensile loads. However, it's vital to acknowledge that in actual anatomical scenarios, opposing forces between the mandible and maxilla encompass multidirectional dimensions, not merely vertical and horizontal. In light of these considerations, it is prudent to interpret the numerical findings from this study with a view toward more comprehensive anatomical evaluations. Alternatively, a clinical study might be

Formatted: Font: 9 pt

Formatted: Font: (Default) Arial, 12 pt

Formatted: Numbered + Level: 1 + Numbering Style: 1, 2, 3, … + Start at: 5 + Alignment: Left + Aligned at: 0.25" + Indent at: 0.5"

Formatted: Font: 9 pt

Formatted: Font: 9 pt

Formatted: Right: 0.25"

Formatted: Position: Horizontal: Right, Relative to: Margin, Vertical: 0", Relative to: Paragraph, Wrap Around

envisaged to validate the clinical implications of the study's findings. In the future, investigations should delve into the tensile strength exhibited when models are displaced in multiple directions, aiming to provide a more comprehensive and robust understanding of maxillo-mandibular fixation mechanics in complex clinical settings.

Acknowledgments: The authors could like to acknowledge Mr. Thammasak Vimonkiattikun for his expertise and assistance in the designing and milling process of the customized dental models and screws.

Author Contributions: Conceptualization, S.P. and S.V.; methodology, S.P. and S.V.; validation, S.P. and S.V.; formal analysis, S.P. and S.V.; investigation S.P., data curation, S.P.; writing- original draft preparation S.P.; writing-reviewing and edition, S.P. and S.V. All authors have read and agreed to the published version of the manuscript.

Funding: This research received no external funding.

Institutional Review Board Statement: Not applicable.

Informed Consent Statement: Not applicable.

~~**Acknowledgments:** The authors could like to acknowledge Mr. Thammasak Vimonkiattikun for his expertise and assistance in the designing and milling process of the customized dental models and screws.~~

Conflicts of Interest: The authors declare no conflict of interest.

Formatted: Font: (Default) Arial, 9 pt, Bold

Formatted: Normal, Left, Indent: Left: 0", First line: 0", Line spacing: Double

Formatted: Font: 9 pt

Formatted: Normal, Line spacing: Double

Formatted: Font: (Default) Arial, 9 pt

Formatted: Font: (Default) Arial

Formatted: Font: (Default) Arial, 9 pt

Formatted: Font: (Default) Arial

Formatted: Font: (Default) Arial, 9 pt

Formatted: Font: (Default) Arial

Formatted: Font: (Default) Arial, 9 pt

Formatted: Font: (Default) Arial

Formatted: Font: (Default) Arial, 9 pt

Formatted: Font: (Default) Arial

Formatted: Font: (Default) Arial, 9 pt

Formatted: Font: (Default) Arial

Formatted: Right: 0.25"

Formatted: Position: Horizontal: Right, Relative to: Margin, Vertical: 0", Relative to: Paragraph, Wrap Around

References

1. Cornelius C-P, Ehrenfeld M. The use of MMF screws: Surgical technique, indications, contraindications, and common problems in review of the literature. Craniomaxillofacial Trauma & Reconstruction. 2010;3(2):55–80. doi:10.1055/s0030-1254376
2. Smith AT. The use of orthodontic chain elastic for temporary intermaxillary fixation. British Journal of Oral and Maxillofacial Surgery. 1993;31(4):250 1.doi:10.1016/0266-4356(93)90150-u

Formatted: Font: 9 pt

Formatted: Font: (Default) Arial

Formatted: Normal, Line spacing: Double

Formatted: Font: (Default) Arial

Formatted: Normal, Line spacing: Double

Formatted: Font: (Default) Arial

Formatted: Normal, Line spacing: Double

Formatted: Font: (Default) Arial, 9 pt

Formatted: Space Before: 0 pt, After: 0 pt, Line spacing: Double

Formatted: Font: 9 pt

Formatted: Font: (Default) Arial, 9 pt

Formatted: Line spacing: Double

Formatted: Right: 0.25"

Formatted: Position: Horizontal: Right, Relative to: Margin, Vertical: 0", Relative to: Paragraph, Wrap Around

3. Manjrekar S, Joshi A. Use of mandibulo-maxillary fixation screws for the treatment of maxillofacial fractures. Dentistry. 2017;7(12). doi:10.4172/2161-1122.1000467
4. Qureshi A, Reddy U, Warad N, Badal S, Jamadar A, Qurishi N. Intermaxillary fixation screws versus Erich arch bars in mandibular fractures: A comparative study and review of literature. Annals of Maxillofacial Surgery. 2016;6(1):25.doi:10.4103/2231-0746.186129
5. Minami R, Morill L, Weber J. Intermaxillary fixation with orthodontic brackets bonded to teeth. Plastic and Reconstructive Surgery. 1974;54(4):492–4. doi:10.1097/00006534-197410000-00022
6. Khajehahmadi S, Rahpeyma A. Force relaxation of 3/16 inch heavy orthodontic latex elastics used in maxillofacial trauma in simulated jaw fracture situation. Dental Hypotheses. 2014;5(4):146. doi:10.4103/2155-8213.140592
7. Kanitkar R, Kumbhare D. Role of orthodontic elastics in the oral & maxillofacial surgical procedures. Journal of Advanced Medical and Dental Sciences Research. 2020;8(2). doi:10.21276/jamdsr
8. Hernández-Alfaro F, Rosemberg V, Masià-Gridilla J, Valls-Ontañón A. Improving accuracy of the intermediate splint in substantial intermaxillary sagittal discrepancies using an extra anterior anchorage point: Technical note. Medicina Oral Patología Oral y Cirugia Bucal. 2021; doi:10.4317/medoral.24089
9. Perez DE, Liddell A. Controversies in orthognathic surgery. Oral and Maxillofacial Surgery Clinics of North America. 2017;29(4):425–40.doi:10.1016/j.coms.2017.07.008
10. Bouya S, Balouchi A, Rafiemanesh H, Amirshahi M, Dastres M, Moghadam MP, et al. Global prevalence and device related causes of needle stick injuries among health care workers: A systematic review and meta-analysis. Annals of Global Health. 2020;86(1):35. doi:10.5334/aogh.2698

Formatted: Right: 0.25"

Formatted: Position: Horizontal: Right, Relative to: Margin, Vertical: 0", Relative to: Paragraph, Wrap Around

11. Erkut Yilridum, John P. Devincenzo; Maximum Opening and Closing Forces Exerted by Diverse Skeletal Types. *Angle Orthod* 1 July 1971; 41 (3): 230–235. doi: https://doi.org/10.1043/0003-3219(1971)041<0230:MOACFE>2.0.CO;2
12. Brunton PA, Loch C, Waddell JN, Bodansky HJ, Hall R, Gray A. Estimation of jaw-opening forces in adults. Orthodontics & Craniofacial Research. 2017;21(1):57–62. doi:10.1111/ocr.12215
13. Das B, Wang Y. Isometric pull-push strengths in workspace: 1. Strength Profiles. International Journal of Occupational Safety and Ergonomics. 2004;10(1):43–58. doi:10.1080/10803548.2004.11076594

1. Cornelius, C. P.; Ehrenfeld, M., The Use of MMF Screws: Surgical Technique, Indications, Contraindications, and Common Problems in Review of the Literature. *Craniomaxillofac Trauma Reconstr* **2010,** *3* (2), 55-80.
2. Smith, A., The use of orthodontic chain elastic for temporary intermaxillary fixation. *British Journal of Oral and Maxillofacial Surgery* **1993,** *31* (4), 250-251.
3. Manjrekar, S.; Joshi, A., Use of Mandibulo-Maxillary Fixation Screws for the Treatment of Maxillofacial Fractures. *Dentistry* **2017,** *7* (12).
4. Qureshi, A. A.; Reddy, U. K.; Warad, N. M.; Badal, S.; Jamadar, A. A.; Qurishi, N., Intermaxillary fixation screws versus Erich arch bars in mandibular fractures: A comparative study and review of literature. *Ann Maxillofac Surg* **2016,** *6* (1), 25-30.
5. Minami, R. T.; Morrill, L. R.; Weber Jr, J., Intermaxillary fixation with orthodontic brackets bonded to teeth. *Plastic and reconstructive surgery* **1974,** *54* (4), 492-494.
6. Rahpeyma, A.; Khajehahmadi, S., Force relaxation of 3/16 inch heavy orthodontic latex elastics used in maxillofacial trauma in simulated jaw fracture situation. *Dental Hypotheses* **2014,** *5* (4), 146.
7. Kanitkar, R.; Kumbhare, D.; Francis, M.; Tiwari, R. V. C.; Kumar, S.; Wasan, B.; Tiwari, H., Role of Orthodontic Elastics in the Oral & Maxillofacial Surgical Procedures. *Journal of Advanced Medical and Dental Sciences Research* **2020,** *8* (2).

Formatted: Font: (Default) Arial, 9 pt

Formatted: Normal, Line spacing: 1.5 lines

Formatted: Font: (Default) Arial, 14 pt, Font color: Auto

Formatted: Justified, Indent: Left: 0", First line: 0", Line spacing: 1.5 lines, Adjust space between Latin and Asian text, Adjust space between Asian text and numbers

Formatted: Font: (Default) Arial, 8 pt

Formatted: Font: (Default) Arial, 8 pt, Font color: Red

Formatted: Right: 0.25"

Formatted: Position: Horizontal: Right, Relative to: Margin, Vertical: 0", Relative to: Paragraph, Wrap Around

8. Hernandez-Alfaro, F.; Rosemberg, V.; Masia-Gridilla, J.; Valls-Ontanon, A., Improving accuracy of the intermediate splint in substantial intermaxillary sagittal discrepancies using an extra anterior anchorage point: technical note. *Med Oral Patol Oral Cir Bucal* **2021,** *26* (2), e151-e155.

9. Perez, D. E.; Liddell, A., Controversies in orthognathic surgery. *Oral and Maxillofacial Surgery Clinics* **2017,** *29* (4), 425-440.

10. Bouya, S.; Balouchi, A.; Rafiemanesh, H.; Amirshahi, M.; Dastres, M.; Moghadam, M. P.; Behnamfar, N.; Shyeback, M.;Badakhsh, M.; Allahyari, J.; Al Mawali, A.; Ebadi, A.; Dezhkam, A.; Daley, K. A., Global Prevalence and Device Related Causes of Needle Stick Injuries among Health Care Workers: A Systematic Review and Meta-Analysis. *Ann Glob Health* **2020,** *86* (1), 35.

11. Yildirim, E., Maximum opening and closing forces exerted by diverse skeletal types. *Journal of Istanbul University Faculty of Dentistry* **1970,** *5* (2), 121-128

12. Brunton, P. A.; Loch, C.; Waddell, J. N.; Bodansky, H. J.;Hall, R.; Gray, A., Estimation of jaw-opening forces in adults. *Orthod Craniofac Res* **2018,** *21* (1), 57-62.

13. Das, B.; Wang, Y., Isometric pull-push strengths in workspace: 1. Strength profiles. *Int J Occup Saf Ergon* **2004,** *10* (1), 43-58.

Formatted: Justified, Line spacing: 1.5 lines, Adjust space between Latin and Asian text, Adjust space between Asian text and numbers

Formatted: Justified, Indent: Left: 0", First line: 0", Line spacing: 1.5 lines, Adjust space between Latin and Asian text, Adjust space between Asian text and numbers

Formatted: Font: (Default) Arial, 8 pt

Formatted: Font: (Default) Arial, 11 pt

Formatted: Font: (Default) Arial, 8 pt

Formatted: Font: (Default) Arial, 11 pt

Formatted: Font: (Default) Arial, 8 pt

Formatted: Font: (Default) Arial, 11 pt

Formatted: Font: (Default) Arial, 8 pt

Formatted: Font: (Default) Arial, 11 pt

Formatted: Font: (Default) Arial, 8 pt

Formatted: Font: (Default) Arial, 11 pt

Formatted: Justified, Line spacing: Double, Adjust space between Latin and Asian text, Adjust space between Asian text and numbers

Formatted: Centered, Line spacing: 1.5 lines, Adjust space between Latin and Asian text, Adjust space between Asian text and numbers

Formatted: Justified, Line spacing: Double, Adjust space between Latin and Asian text, Adjust space between Asian text and numbers

Formatted: Font: (Default) Arial, 8 pt

Formatted: Right: 0.25"

Formatted: Position: Horizontal: Right, Relative to: Margin, Vertical: 0", Relative to: Paragraph, Wrap Around

Rebuttal Letter

August 16th 2023,

Dear Editor and Reviewers,

On behalf of all authors, we are grateful for your helpful in-depth feedback on our manuscript. We hope that you consider all raised points to be adequately addressed by our revised manuscript as described below and that the revised manuscript qualifies for publication.

Kind Regards,

Surakit Visutiwattanakorn

Editor Comments

1. Abstract: Condense the first three sentences into one to provide a concise overview. Focus more on explaining the meaning of the findings and their clinical implications.

   The abstract has been revised to provide a more concise overview of the manuscript.

2. General format: Ensure that the manuscript follows the PLOS ONE format. Please consult the information for authors to ensure compliance.

   The manuscript has been edited to follow the PLOS ONE format as written on the "information for authors" page.

3. Introduction: Although the introduction provides a comprehensive overview of intermaxillary fixation (IMF) or maxillo-mandibular fixation (MMF), there are areas for improvement:

   3.1) Clearly state the research problem or objective in a concise manner. Highlight the specific research question or knowledge gap that the study aims to address.

   3.2) Establish the need for further investigation or improvement by discussing the limitations, challenges, or evidence gaps associated with current techniques. Justify the need for the study by providing relevant information from the literature.

   3.3) Elaborate on the advantages and limitations of orthodontic latex elastics as an alternative to wire ligatures. Briefly discuss the advantages and disadvantages of different fixation methods to enhance the reader's understanding of the research context.

   3.4) Restructure the introduction to ensure a logical flow and organization of information. Improve transitions between ideas to present a coherent and logical progression of concepts.

   The introduction has been rewritten and involves all the changes recommended by the editor and reviewers. The research problem and objectives have been clearly stated so that the reader clearly understands the knowledge gap and what this study aims to address.

4. Methods: While the methods provide sufficient experimental details, include information on how the model simulates clinical situations. If there is clinical validation, provide relevant details. If not, discuss how the results can be applied to clinical patients.

   The materials and methods have been reformatted to better explain how the custom-made models simulate real-life scenarios. The purpose of the study has been stated to further discuss how the results can be applied.

5. Discussion and Conclusion: Separate the Discussion and Conclusion sections. The Discussion should follow this format: 4.1) highlight important results, 4.2) compare the results with previous studies, 4.3) discuss the limitations of the study, and 4.4) indicate clinical implications and suggest future studies. Move Figure 7 from the Discussion section to the Results section.

   The discussion and conclusion sections have been separated as recommended. Figure 7 has been moved and re-cited within the results section.

6. Conclusion: Provide a clear and direct summary of the results in terms of addressing the hypothesis. Since the manuscript lacks a clear clinical problem and hypothesis, ensure the conclusion is based on the obtained results.

   The conclusion has been totally re-written as per the editors and reviewer's recommendation.

Journal Requirements

1. Please ensure that your manuscript meets PLOS ONE's style requirements, including those for file naming.

   I have re-formatted my manuscript according the PLOS ONE's style requirements. Please let me know if any additional changes need to be made.

2. PLOS requires an ORCID iD for the corresponding author in Editorial Manager on papers submitted after December 6th, 2016. Please ensure that you have an ORCID iD and that it is validated in Editorial Manager.

   I have updated my profile to include my ORCID iD in the Editorial Manager Portal.

3. We note that Figures 1, 2, 3, and 4 in your submission contain copyrighted images. All PLOS content is published under the Creative Commons Attribution License (CC BY 4.0), which means that the manuscript, images, and Supporting Information files will be freely available online, and any third party is permitted to access, download, copy, distribute, and use these materials in any way, even commercially, with proper attribution.

   Figures 1-4 are original photographs taken by Sudeep Pawa during the experimentation phase of this study. Is it still necessary to have the copyright paperwork for this situation?

Reviewer #1: To begin with
- English language editing services very much needed for manuscript.
- This is a CAD based design which was utilised for this invitro study . Wat was the printing material utilised for this study????
- Please provide justifications and citations to say the model material doesn't interferes with testing results.
- Please justify your sample size
- Please add more correct citations under discussion. Total references of 13 for an original article is unacceptable.
- Wat is new in this study when compared to previously published articles on similar topic.
- Please provide relevant citations to your materials and methods section.
- Your study is on tensile load only. Wat about shear loads acting on mandible at time of Fixation. Anything included in your study ???

Reply to Reviewer # 1

We sincerely appreciate your insightful and thorough review of our manuscript. The manuscript has undergone grammatical revisions. As detailed within, the customized models were created through the utilization of Computer-Aided Design (CAD) and subsequently fabricated via Computer-Aided Manufacturing (CAM). These models were constructed utilizing Stainless Steel as the chosen material. While material selection lacks specific justification, it is worth noting that material properties did not influence this study's scope, which solely focused on analyzing Tensile Loads causing separation between the two models.

In the context of sample size, considering our status as a pilot study embarking on the pioneering examination of tensile loads in Maxillo-Mandibular Fixation, an initial pilot study was conducted. Sample size determination was carried out employing Stata/SE 15.1 for power analysis concerning one-way analysis of variance. This calculation incorporated a statistical significance level of 0.001, alongside a power value of 0.99, drawing on insights garnered from the pilot study's data.

Given the absence of prior experimental investigations into tensile loads encompassing varied patterns during maxillo-mandibular fixation, it is important to acknowledge that direct comparisons with previous research cannot be made. Furthermore, with regards to shear loads impacting the mandible, the study was intentionally designed to explore vertical (unidirectional) forces exclusively. It is acknowledged that this design choice represents a limitation within our study.

Reviewer #2: Nice preclinical project, not without its limitations as noted. Well written and documented. A couple of minor suggestions:
1. although the outcomes will not change, I would recommend indicating the parameters that were tested and to what level they were tested when completing the power analysis.
2. Not being an OMS or orthodontist and being familiar with the up to date state of MMF and arrangement of wires and elastics commonly used in MMF, it might help the reader to know what currently is the standard configuration that is taught and used in clinical practice to achieve fixation. If internal fixation is used is external fixation even needed? The OMS department in my current institution rarely in orthognatic cases uses external fixation once the internal fixation is placed. I would make comment of the current gold standard, and in what cases would you prefer external vs internal fixation. Additionally, I would also maybe make more comment of the consequences of inadequate fixation in the introduction.
3. Discuss experimental model limitations in greater detail and contrasting to what is done clinically- while MM bone fixation screws may be used (I am guessing not usually one for every tooth as used in this model), as indicated orthodontic brackets +- MMF screws are more commonly used. Why was one screw used for every tooth? What was the rationale? Why were orthodontic brackets not used if this is more commonly used in clinical practice? If repeating or extending this experimental model to study the effects of lateral forces, I would suggest integrating orthodontic brackets into the model (if you are able to find a way to bond to the custom 'dental model') to better represent what may be done clinically. This may be a point for discussion in the limitations of this preclinical model, along with discussion of any potential differences in the size of the custom fixation screw that was used in this study, to what it commonly available on the market (they seem quite large in diameter and not what may be used clinically). Granted this is a preclinical study with obvious limitations, clinical correlation and deeper discussion on model limitations is helpful for the reader as if products/screws that are used are not commercially available or the set up is not as it would be in a real situation then the results have minimal clinical meaning.
Other than that, nice project. I would be interested to see as you mentioned how it would fare up with lateral movements which if the patient is a bruxer may be the time when potentially higher forces are generated and this could impact the fixation.

Reply to Reviewer # 2

We sincerely appreciate your insightful and thorough review of our manuscript. The study has taken steps to enhance reader comprehension by rephrasing the study's parameters and detailing confidence levels or p-values. Regarding the established benchmark for achieving satisfactory Maxillomandibular Fixation (MMF), it's important to note that both techniques continue to be employed globally without a definitive consensus on a superior method. Concerning internal and external fixation, External Fixation (MMF) is frequently employed to facilitate precise and anatomical internal fixation. In the postoperative phase, external fixation serves to guide occlusion training in numerous instances. The introduction has now been supplemented with information on the ramifications of insufficient MMF.

For the purpose of this pilot study and to mitigate variables influencing outcomes, the authors opted for the use of screws instead of orthodontic brackets. This strategic choice eliminated potential factors tied to bracket bond strength, which would have been pertinent had brackets been employed. Clear delineations have been provided about the study's limitations, along with suggestions for future research based on this study's findings

Reviewer #3: The study is well designed and the manuscript is also acceptable for publication. However, it would be better if the clinical implication(s) of the in vitro findings are highlighted. The article seems to lack novelty.

Reply to Reviewer # 3

We sincerely appreciate your insightful and thorough review of our manuscript. Clinical implications of this in vitro study have been added. We hope that the changes are sufficient to qualify for publication.
