# Supplementary material for: An in vitro study in separating tensile loads during maxillo-mandibular fixation using wire and/or elastics
Source: PLoS One. 2024 Mar 15;19(3):e0300481. doi: 10.1371/journal.pone.0300481 (PMC10942067; doi:10.1371/journal.pone.0300481)
Supplement: Attachment — Submitted filename: Rebuttal Letter.docx [file pone.0300481.s005.docx]

Rebuttal Letter

November 20^th^ 2023,

Dear Editor and Reviewers,

On behalf of all authors, we are grateful for your helpful in-depth feedback on our manuscript. We hope that you consider all raised points to be adequately addressed by our revised manuscript as described below and that the revised manuscript qualifies for publication.

Kind Regards,

Surakit Visutiwattanakorn

**Reviewer #1**: I still see Abstract is not a structured one . Please provide a structured abstract. Please provide all data related to this study in a repository or a supplemental file . I meant all those files which had not been mentioned in the revised manuscript.

**Reply to Reviewer #1:** Thank you for your kind acknowledgment of our comprehensive review of your manuscript. We are glad to inform you that the abstract has been meticulously re-written and restructured to enhance clarity, facilitating a more effective understanding of the study's purpose and results.Additionally, we have included files that contain data from the Instron 5566 Universal Testing Machine ensuring transparency and completeness, in this research.

**Reviewer #2**: I question the use, discussion and relevance of reference #13 on line 234. It appears the study referenced is in relation to a persons ability to pull down on a level with their arm in a seating and standing position. I fail to see how this study is relevant to the discussion of forces that could potentially be applied in the oral region. I am sure there are more appropriate references to discuss, allowing for a clinical correlation/relevance. This may also enlighten us as to if doing the combined fixation is the way to go, or is the additional stability achieved in this model irrelevant and so standard wire fixation is all that is needed?
- another clinical relevance note - forces from the elastics degrade over time and so if this type of fixation is needed for some time, elastics will need to be replaced.
- I would move the discussion on model limitations to the end of the discussion section and not in the conclusions section

**Reply to Reviewer #2:**  We truly value your thorough examination of our manuscript. Reference #13 was carefully selected as a benchmark, in our study due to its relevance in assessing the forces applied to the maxillo unit during intraoperative reduction and fixation. Our main goal was to present the findings from our in vitro experiments regarding the forces needed to cause separation of models during fixation. It is essential to clarify that our investigation focused on the intraoperative phase, examining the immediate effects of intermaxillary fixation, rather than addressing potential degradation of elastics over time, which may be a significant factor in the post-operative period. Our aim was to offer a numerical representation derived from in vitro experiments, intending to establish a reference point for future clinical studies. The discussion on model limitations has also been moved to the discussion section.

**Reviewer #3**: Dear doctor, despite your efforts, your experimental model has several inadequacies, that make the results invalid for clinical interpretation. The experimental model is merely indicating the tensile properties of various materials and is not even a faint replication of the clinical situation. The results obtained from this study are insignificant as long as they do not have any strong correlation with the clinical situation. Combining two materials will always give the concluded result. Please provide a strong rationale for your study, to justify its publication.

**Reply to Reviewer #3:** Given the absence of prior experimental investigations into tensile loads encompassing varied patterns during maxillo-mandibular fixation, it is important to acknowledge that direct comparisons with previous research cannot be made. Furthermore, with regards to shear loads impacting the mandible, the study was intentionally designed to explore vertical (unidirectional) forces exclusively. It is acknowledged that this design choice represents a limitation within our study. For the purpose of this pilot study and to mitigate variables influencing outcomes, the authors opted for the use of screws instead of orthodontic brackets. This strategic choice eliminated potential factors tied to bracket bond strength, which would have been pertinent had brackets been employed. Clear delineations have been provided about the study's limitations, along with suggestions for future research based on this study's findings. Our goal was to provide an in vitro numerical representative value that would serve as a reference for future clinical studies.
